# Non-programmed transcriptional frameshifting is common and highly RNA polymerase type-dependent

**DOI:** 10.1186/s12934-018-1034-4

**Published:** 2018-11-24

**Authors:** Dawid Koscielniak, Ewa Wons, Karolina Wilkowska, Marian Sektas

**Affiliations:** Department of Microbiology, Faculty of Biology, University of Gdansk, Wita Stwosza 59, 80-308 Gdansk, Poland

**Keywords:** Recombinant proteins, *E. coli* RNAP, T7 bacteriophage RNAP, Expression system, GFP reporter, Transcriptional slippage, Indel errors

## Abstract

**Background:**

The viral or host systems for a gene expression assume repeatability of the process and high quality of the protein product. Since level and fidelity of transcription primarily determines the overall efficiency, all factors contributing to their decrease should be identified and optimized. Among many observed processes, non-programmed insertion/deletion (indel) of nucleotide during transcription (slippage) occurring at homopolymeric A/T sequences within a gene can considerably impact its expression. To date, no comparative study of the most utilized *Escherichia coli* and T7 bacteriophage RNA polymerases (RNAP) propensity for this type of erroneous mRNA synthesis has been reported. To address this issue we evaluated the influence of shift-prone A/T sequences by assessing indel-dependent phenotypic changes. RNAP-specific expression profile was examined using two of the most potent promoters, P_araBAD_ of *E. coli* and φ10 of phage T7.

**Results:**

Here we report on the first systematic study on requirements for efficient transcriptional slippage by T7 phage and cellular RNAPs considering three parameters: homopolymer length, template type, and frameshift directionality preferences. Using a series of out-of-frame *gfp* reporter genes fused to a variety of A/T homopolymeric sequences we show that T7 RNAP has an exceptional potential for generating frameshifts and is capable of slipping on as few as three adenine or four thymidine residues in a row, in a flanking sequence-dependent manner. In contrast, bacterial RNAP exhibits a relatively low ability to baypass indel mutations and requires a run of at least 7 tymidine and even more adenine residues. This difference comes from involvement of various intrinsic proofreading properties. Our studies demonstrate distinct preference towards a specific homopolymer in slippage induction. Whereas insertion slippage performed by T7 RNAP (but not deletion) occurs tendentiously on poly(A) rather than on poly(T) runs, strong bias towards poly(T) for the host RNAP is observed.

**Conclusions:**

Intrinsic RNAP slippage properties involve trade-offs between accuracy, speed and processivity of transcription. Viral T7 RNAP manifests far greater inclinations to the transcriptional slippage than *E. coli* RNAP. This possibly plays an important role in driving bacteriophage adaptation and therefore could be considered as beneficial. However, from biotechnological and experimental viewpoint, this might create some problems, and strongly argues for employing bacterial expression systems, stocked with proofreading mechanisms.

**Electronic supplementary material:**

The online version of this article (10.1186/s12934-018-1034-4) contains supplementary material, which is available to authorized users.

## Background

Random fluctuation in promoter activity influenced by intrinsic and extrinsic factors causing cell-to-cell variability in the mRNA level during expression of an individual gene is referred to as genetic noise [1–4]. It creates a selective advantage for competing strains by allowing response to changes in the environment. More specifically, phenotypic variability in clonal populations of microbial cells can be generated by various epigenetic events [5–8]. Changes in the gene expression pattern are triggered by factors which alter the genetic record without affecting the primary DNA sequence [9–12]. Among them, the programmed transcriptional realignment contributing to the site specific RNA editing appears to have the most influential impact on gene expression and regulation [11, 13–15]. However, our knowledge on non-programmed, “accidental” nucleotide indel errors in slippage-prone A/T tracts, remains incomplete, and thus its real impact on gene expression profile may be underestimated. Recently, an incredible propensity for slippage on many homopolymeric regions within a given gene found for T7 and *E. coli* RNAPs resulted in a production of a broad spectrum of protein variants with some amino-acid alterations [16]. Productive transcriptional slippage at such sites involves unwinding-rewinding of the RNA:DNA hybrid which is not sensed by RNAP active site and thereby does not lead to the enzyme’s backtracking and mRNA correction. Dissociation of 8–9 nucleotide duplex and re-pairing is much more plausible within a series of identical nucleotides than a random mixed sequence. According to the thermodynamic model many microscopic base slippage events can lead to local RNA displacements [17, 18]. Decrease in fidelity may result from poor stability of the 3′-proximal part of the hybrid which occurs at A–U rich tracts. Long mononucleotide A/T stretches destabilize and realign RNA:DNA hybrid, contributing to a slip of RNAP on the DNA template. Studies focused on influence of the A/T homopolymeric regions on helix structures revealed their unusual properties, modifying the normal conformation of B-DNA model [19–23]. In this light the poly(A/T) tracts are considered as structures potentially promoting a less accurate transmission of genetic information. Accordingly, the poly(A) homopolymer regions are more tightly wound (10.1 ± 0.1 base pairs per turn). The adenine N-6 amines of the one strand and thymine O-4 of the subsequent base pair of the opposite strand can form cross-chain hydrogen bonds which stabilize a large propeller twist in the A–T base pairs. As was demonstrated, rA:dT polypurine hybrids have a severely distorted structure, encompassing altered base pairing (weakened pairing, mispairing and unpairing) which leads to an unzipped structure, found in free RNA:DNA hybrids as well as in a complex with HIV reverse transcriptase [24, 25]. Molodtsov et al. [26] regarded poly(U/A) structural abnormalities as the prerequisite for the transcription termination by T7 and SP6 RNAPs. A plausible model of the nucleotide misincorporation during T7 phage, yeast, *Thermus thermophilus* and *E. coli* RNAP transcription is the template strand misalignment by temporary flipping-out of a base [17, 27–29].

Indel type of transcriptional errors may be considered as ambiguous. One on hand, they are detrimental to efficiency of gene expression, but on the other hand under certain conditions they can also be beneficial for cell physiology. The level of expression and multispecificity of numerous genes (phenotypic variation) may be regulated in this way [6, 30, 31]. Moreover, transcriptional slippage has significant potential to restore the wild-type phenotype of indel mutant genes [10, 16, 32–36]. In fact, epigenetic effects caused by transcriptional slippage have been found during all steps of the transcription process in *E. coli*, particularly at homopolymeric stretches of A/T, which appear to be hot-spots for subsequent phenotypic changes [16, 34, 37–40].

In this work, we are presenting comparative analysis of the transcriptional slippage propensity of the two most used RNA polymerases: *E. coli* and T7 bacteriophage, representing two different enzyme families [41]. In our study, we used a set of poly(A/T) sequence variants translationally fused to downstream located out-of-frame green fluorescent protein gene (*gfp*) of *Aequorea victoria*, which allowed to monitor the most frequent, single nucleotide insertion and deletion in mRNA. Depending on the specific sequence context we noticed up to several fold greater slippage ability of T7 RNAP than the host polymerase. These enzymes also differ in their preference towards homopolymeric tracts. T7 RNAP is similarly error prone on both poly(A/T)s, while the host polymerase is conspicuously more liable to slippage on poly(T)s. Obtained results, despite addressing only one aspect of mRNA synthesis fidelity, reflect two interesting and important distinct survival strategies. In case of highly organized bacterial cells the aim is to avoid any unfavorable mutations, while for bacteriophage the changes may turn out to bear an adaptive advantage.

## Materials and methods

### Bacterial strains and plasmids

*Escherichia coli* (*E*. *coli*) DH10B (F^–^ λ^–^ *mcr*A Δ(*mrr*-*hsd*RMS-*mcr*BC) Φ80*lac*ZΔM15 Δ*lac*X74 *rec*A1 *end*A1 *ara*D139 Δ(*ara leu*) 7697 *gal*U *gal*K *rps*L *nup*G) and ER2566 (F^–^ λ^–^ *fhu*A2 [*lon*] *omp*T *lacZ*::T7 gene *1 gal sulA*11 Δ(*mcr*C-*mrr*)114::IS*10* R(*mcr*-73::miniTn*10*-Tet^S^)2 R(*zgb*-210::Tn*10*-Tet^S^) *end*A1 [dcm]) both from New England Biolabs were used as a cloning and/or expressing host. Plasmid pGreenTIR was a source of enhanced fluorescence eGFP variant (F64L/S65T) [42]. *E. coli* cells were grown in Luria–Bertani (LB) broth or agar [43] with antibiotics (Sigma) at the following concentrations: ampicillin (Ap), 100 μg ml^−1^; chloramphenicol (Cm), 25 μg ml^−1^; kanamycin (Km), 50 μg ml^−1^; and tetracycline (Tet) 15 μg ml^−1^, chlorotetracycline (cTc) 20 μg ml^−1^, bicyclomycin (BMC, Cayman Chemicals) 5 – 100 μg ml^−1^. Plasmids used in this work (Additional file 1: Table S1) were introduced into the cells by a standard chemical transformation procedure [43]. For each experiment freshly transformed cell were used. After reaching mid-log phase, the appropriate cell culture was supplemented with l-arabinose (Sigma) or IPTG (isopropyl-β-d-1-thiogalactopyranoside, Sigma) at a concentration at 0.1% and 1 mM, respectively, and the cells were harvested after 1 h of additional incubation.

### Strain construction

*Escherichia coli* MC1061Δ*greAgreB* strain was constructed by two-step P1*vir* transduction [43] with the following donors: CF15971 [44] as a source of Δ*greA*::*cat*, and CF15977 as a source of Δ*greB*::*kan*. Both strains were obtained from Dr. K. Potrykus.

### Plasmids construction

Construction of plasmids employed combination of PCR and subcloning techniques to provide variants of the *mboIIM2* gene, *mboIIM2*-*gfp* or poly(AT)-*gfp* as translational fusion genes inserted into pBAD24 [45] or pET24a (Novagene, USA) expression vectors, containing the P_araBAD_ or φ10 of T7 phage promoter, respectively. All details concerning plasmid constructs are given in Additional file 1: Table S1.

### Genetic techniques

Standard protocols [43] and kits were used for purification of plasmid DNA (A&A Biotechnology, Poland), DNA digestion with restriction endonucleases, DNA ligation with T4 DNA ligase, PCR techniques with PfuPlus DNA polymerase (all from Eurx-Gdansk, Poland), as well as for DNA sequencing of the *mboIIM2* mutated derivatives (Genomed, Poland).

### Single and multiple site-directed mutagenesis

Indel variants of DNA methyltransferase *mboIIM2* from *Moraxella bovis* ATCC 10900 [46] were constructed by introducing nucleotide deletion/insertion in reverse primers. Supplementary tables include a list of plasmids with details of their construction and oligonucleotides used (Additional file 1: Tables S1 and S2, respectively). Site-specific mutagenesis using PCR was carried by high fidelity PfuPlus DNA polymerase (Eurx-Gdansk, Poland) according to the manufacturer’s instructions (50 ng of plasmid template was added to a 50-μl PCR mix). Appropriate plasmid templates were PCR-amplified and then 1 μl (10 u) of the DpnI enzyme (Fermentas) was added directly to reactions to eliminate the parental plasmid DNA. Following a-1.5-h incubation at 37 °C, the DNA products were resolved in agarose gels, appropriate bands were cut out and aliquots containing purified DNA were transformed into DH10B competent cells. All plasmid modifications were confirmed by Sanger DNA sequencing using the BigDye Terminator v3.1 (Applied Biosystems, USA) (Genomed, Poland).

### Segmental analysis of *mboIIM2* gene transcripts decay by real-time qRT-PCR and northern blotting detection

Cellular RNA collected for RT-qPCR and northern blot analyses was extracted using the Total RNA Mini Kit (A&A Biotechnology Poland) according to the manufacturer’s instructions. RNA from individual sample extraction (8 μl out of 80 μl total RNA) was used in each 20 μl reaction for cDNA synthesis (Thermo Scietific). The specific forward and reverse primers used for testing *mboIIM2* variants mRNA and internal controls are shown in Additional file 1: Table S2. Eurx Taq DNA polymerase kits (Eurx, Gdansk Poland) were used to prepare the master mix for each sample. Real-time RT-qPCR tested five regions of a transcript covering the whole gene. PCR employed the following cycling parameters: 95 °C for 2 min, followed by 35 cycles of 94 °C for 5 s, 59 °C for 5 s, and 72 °C for 15 s; the melting curve (59–94 °C) program was used for quality control. qRT-PCR was carried out by performing three independent experiments, each in triplicate, in a Lightcycler 2.0 instrument (Roche Diagnostics, Indianapolis, IN, USA) according to the manufacturer’s recommendations by using the SYBR Green real-time PCR kit (Eurx, Gdansk, Poland). In all studies the decay characteristics of the upstream and downstream mRNA segments were normalized to stable reference *cysG* housekeeping gene [47]. The relative fold-change in mRNA ratios were obtained by normalizing each time point data in reference to the earliest measurement [48].

The *mboIIM2* gene variants’ specific transcript profiles were detected by northern blotting. Equal amounts (8 μg) of total RNA from different time intervals were loaded on formaldehyde 1.3% agarose denaturing gel and then transferred onto a nitrocellulose membrane (Zeta-Probe, BioRad) by capillary forces. PCR-produced dsDNA fragment of 380 bp (primers used were Mbo2F and InterR specific to 5′ half of the gene sequence) (Additional file 1: Table S2) was biotin labeled (Biotin-High Prime, Roche) and used for hybridization at 42 °C. Chemiluminescent detection was carried out using streptavidin-HRP Pierce ECL substrate (Thermo Fisher Scientific) followed by exposure on X-ray film.

### Assessment of *mboIIM2* mRNA nucleotide polymorphism by NGS approach

Preparation of cDNA templates (pBADmboIIΔA356) from arabinose induced cells (0.1% for 2 h) was performed exactly as described previously [16]. Similarly, the NGS procedure was performed by Genomed Ltd. (Warsaw, Poland).

### Western blotting

ER2566 cells harboring various pET24a-mboIIM2/gfp plasmids or DH10B carrying pBAD-mboIIM2/gfp plasmids were grown in LB medium at 37 °C and induced with 1 mM IPTG or 0.1% l-arabinose for up to 3 h, respectively. Protein samples (100–200 μl each) were electrophoresed on a 10–12.5% SDS–polyacrylamide gel (PAGE) and electroblotted onto a nitrocellulose membrane (Bio-Rad) using semi-dry transfer system (Pierce G2 Fast Blotter) according to the manufacturer’s instructions (Thermo Scientific). Next, the membrane was blocked for at least 1 h in the PBS buffer (137 mM NaCl, 2.7 mM KCl, 10 mM Na_2_HPO_4_, 2 mM KH_2_PO_4_ pH 7.4) with 5% skimmed milk. The membrane was then probed with rabbit polyclonal anti-M2.MboII [46, 49] or mouse monoclonal anti-GFP (B-2) antibodies (Santa Cruz Biotechnology, USA), diluted 1:2000 and 1:4000, respectively, in TBS-T buffer (50 mM Tris–HCl, 150 mM NaCl, 0.05% Tween 20, pH 7.6) with 5% skimmed milk, for 1.5 h at room temperature. After three washes with TBS-T, the membrane was incubated for 1 h with goat anti-rabbit secondary antibody conjugated with alkaline phosphatase (AP, 1:30,000, Sigma), for 1 h at room temperature, or with a chicken anti-mouse IgG-HRP (horseradish peroxidase, 1:5000, Santa Cruz Biotechnology). The membrane was washed three times and a specific protein was visualized by adding BCIP/NBT solution (Fermentas) or by adding a chemiluminescent substrate solution (Pierce ECL Plus Western Blotting Substrate, Thermo Scientific) and exposed to X-ray film.

### Analysis of the N-terminal amino acid sequence of truncated variants of M2.MboII

N-terminal GFP protein sequence analysis was performed at BioCentrum Ltd. (Krakow, Poland). Sequentially detached phenylthiohydantoin derivatives of amino acids were identified using the Procise 491 (Applied Biosystems, Foster City, USA) automatic sequence analysis system, according to the standard protocol of the manufacturer.

### Quantification of GFP fluorescence

For fluorescence measurements of GFP hybrid proteins, freshly transformed cells were used. Several colonies of each were transferred into 5 ml LB medium and grown until mid-log phase. To induce expression of studied genes IPTG (to 1 mM) or arabinose (to 0.1%) were added, and cells were grown for an additional hour. A total of 400 µl of each cell culture were centrifuged at 3500 rpm for 6 min, supernatant was discarded and the pellet was resuspended in 200 µl of the F buffer (M9 salts; 0.1 mM CaCl_2_; 1 mM MgSO_4_). Fluorescence intensity of the set of poly(A/T)-*gfp* fusions (i.e. eGFP [50, 51]) was quantified using a Varioskan^®^ Flash Spectral Scanning Multimode Reader spectrophotometer (Thermo Scientific) at excitation and emission wavelengths of 485 and 510 nm, respectively, using 96-well black plates (400 μl sample). Samples were assayed in at least three triplicate repetitions. Fluorescence from *gfp*-less cells was taken as background and subtracted from all values. Strains containing in-frame *gfpA*_*6*_*0* and *gfpT*_*6*_*0* fusions were used as positive controls and were taken as 100% [52].

### Fluorescence microscopy

Samples of ER2566 bacterial cultures (0.1 ml) bearing a *gfp*-tagged reporter were studied with fluorescence microscopy. Cell membranes were stained with SynaptoRed C2 (FM4-64) fluorescent dye (Sigma) at a final concentration of 5 μg ml^−1^ for 10 min. The DNA was visualized by staining with DAPI (4,6-diamidino-2-phenylindole) at 1 μg ml^−1^ for 10 min. Samples were then immobilized on 1-mm 1.5% agarose pads dissolved in LB medium and visualized using a Leica DMI4000B microscope fitted with a DFC365FX camera (Leica). The following Leica filter sets were used: N2.1 (for FM4-64), green fluorescent protein (for GFP), and A4 (for DAPI). Images were collected and processed using LAS AF 3.1 software (Leica).

## Results

### T7 RNA polymerase is superior over *E. coli* host polymerase in rescue of a deletion mutation gene

**Fig. 1:**
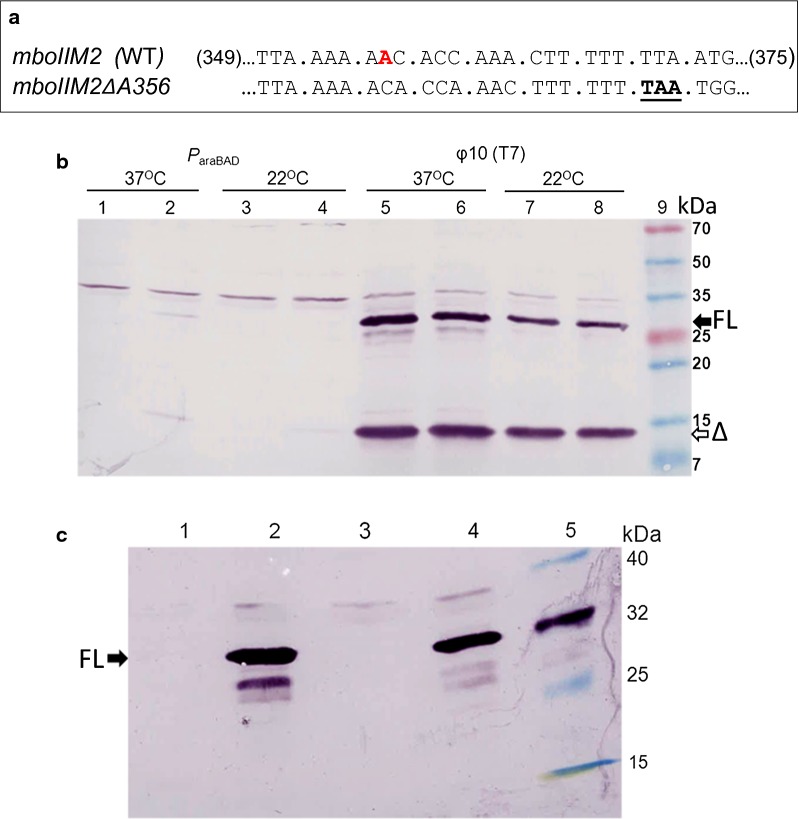
Transcriptional slippage efficiency of *E. coli* and T7 phage RNA polymerases. **a** Details of the relevant sequence of the wild-type and *mboIIM2ΔA356* single nucleotide deletion genes. The position of the A356 nucleotide is marked in red. Premature stop codon is underlined. **b** Level of expression of the *mboIIM2ΔA356* deletion mutant in frame 0 (short product [Δ]—14.5 kDa) and after insertional slippage (full length [FL] product WT—32 kDa), respectively, determined by western blotting. Expression was driven by *E. coli* RNAP (DH10B, lanes 1–4) or T7 RNAP (ER2566, lanes 5–8) from a gene located on pBAD24/T7mboBΔ plasmid at 37 and 22 °C, respectively. Induction of expression was carried out for 3 h with 0.1% l-arabinose (lanes 2 and 4) or 1 mM IPTG (lanes 6 and 8), respectively. Lanes 1, 3, 5 and 7 cell lysates from non-induced cultures. Equal amounts of total protein extracts normalized to OD_600_ were run on 12.5% SDS-PAGE, and immuno detected by using rabbit anti-M2.MboII polyclonal antibody and secondary anti-rabbit-AP. Note, that “leaky” production of M2.MboII proteins under non-inducing conditions (lanes 5 and 7) is due to lack of additional copy of *lacI* repressor gene on the pBAD24 backbone. Lane 9, molecular size prestained protein markers (EUR_x_—Poland). **c** Immunodetection of the wild-type *mboIIM2* gene product generated by the host (DH10B) or T7 RNAP (ER2566), respectively. Appropriate competent cells were transformed with pBAD24/T7mboWTlacI^q^ plasmid and the expression of the gene was induced for 2 h at 37 °C with 0.1% l-arabinose/0.05 mM IPTG (lane 2) or 1 mM IPTG (lane 4), respectively. Lanes 1 and 3, cell lysates from non-induced cultures; lane 5, prestained protein markers, including purified M2.MboII protein

Previously, we had shown that the transcriptional slippage by T7 phage RNAP very effectively rescued single and double nucleotide indel mutations downstream of poly(A/T) regions, scattered over the *mboIIM2* gene [16]. It was manifested by the production of an extra protein in addition to the truncated M2.MboII, i.e. the full-length M2.MboII methyltransferase, as a result of the indel-type mRNA edition. Subsequently, we wanted to compare the transcriptional slippage propensity of the two RNA polymerases distinct in terms of their origin and molecular structure, i.e. multisubunit one from the bacterial cell host (*E. coli*) and the single-subunit one from its parasite, bacteriophage T7 [53]. We constructed a plasmid (pBAD24/T7mboBΔ) carrying the model *mboIIM2ΔA356* gene with the frameshift mutation ΔA356 under independent control of both, *P*_araBAD_ and φ10 promoters [45, 54]. Production of M2.MboII trans-frame variants in *E. coli* cell extracts was monitored by western blotting using anti-M2.MboII antibodies. As expected, after induction of T7 RNAP expression with IPTG, high level of both, the short and the full-length M2.MboII proteins were obtained regardless of the temperature conditions used during culturing of bacteria (Fig. 1a, lanes 6 and 8, respectively). In striking contrast, the production of both M2.MboII variant proteins by the host RNAP following arabinose induction was barely seen, reaching 40-fold lesser level than in the case of T7 RNAP specific expression (Fig. 1a, lanes 2 and 4, respectively). This was quite unexpected, since the *P*_araBAD_ promoter is considered as one of the most powerful in *E. coli*. Indeed, the efficiency of the wild-type *mboIIM2* gene expression was high in both cases (Fig. 1b, lanes 2 and 4, respectively). To exclude the possible influence of a spontaneous mutation which could lower the *P*_araBAD_-dependent expression of the *mboIIM2ΔA356* carrying clones, we subcloned the “less-productive” gene from pBADmboB.4 plasmid into an intact pET24a vector under T7 promoter control, resulting in the pETremboB.4 construct. All randomly chosen recombinants exhibited high level production of truncated and full-length variants after induction of T7 RNAP (Additional file 1: Figure S1, lane 7).

### Intracistronic polarity effect during *mboIIM2ΔA356* gene expression

**Fig. 2:**
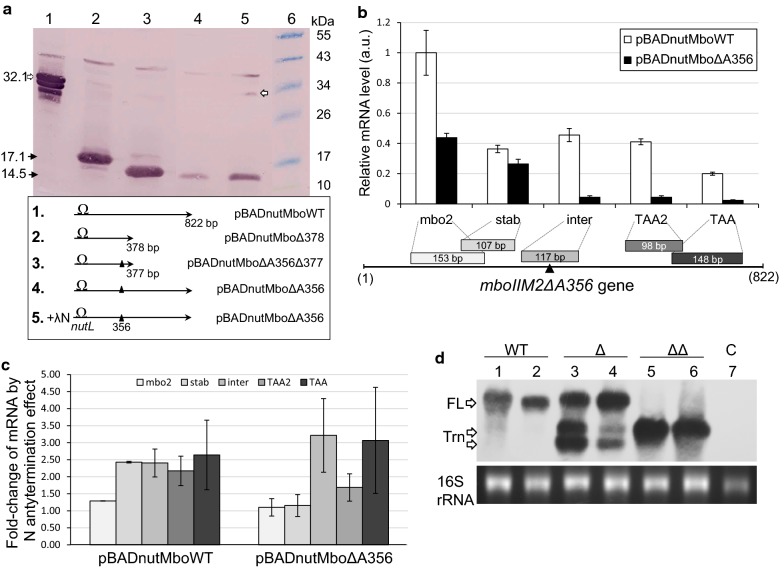
The effect of intracistronic polarity during *mboIIM2ΔA356* expression driven by the host RNAP. **a** Abolition of the polarity effect by reduction of *mboIIM2ΔA356* gene length. Western blot of expressed *mboIIM2* gene mutant variants in DH10B strain in the absence (lanes 1–4) and presence of λ phage N/*nutL* antitermination (lane 5). All cultures were 0.1% l-arabinose induced by 1 h at 37 °C. Equal amounts of total protein extracts from cultures harboring appropriate genes were analyzed after 12.5% SDS–PAGE and immunodetected with anti-M2.MboII antibodies. The schematic structure of genes was depicted below. Lane 6, molecular size markers (Thermo Scientific). Bands of the full-length (32 kDa) and short variants of M2.MboII (14.5 and 17.1 kDa) are marked by arrows. **b** Stability of *mboIIM2* (white) and *mboIIM2ΔA356* (black) gene transcripts analyzed by segmental real-time RT-qPCR. Results are shown as fold-change of expression. Error bars represent standard deviations from three replicates done twice. Location of primers used in RT-qPCR is depicted. **c** Suppression of polarity effect by N/*nutL* antitermination. The relative fold-change of particular segments of transcripts was measured by RT-qPCR and calculated as described in “Materials and methods” section. **d** Northern blot showing the stability pattern of *mboIIM2* (WT), *mboIIM2ΔA356* (Δ) and *mboIIM2ΔA356Δ377* (ΔΔ) gene transcripts in the MC1061 wild-type and CH1828 *rne*-1 cells. MC1061 (lanes 1, 3 and 5) or CH1828 (lanes 2, 4 and 6) were transformed with appropriate plasmids and expression of *mboIIM2* gene variants was induced with 0.1% l-arabinose for 20 min at 42 °C. Top panel: total RNA was subjected to 1.3% agarose-formaldehyde gel electrophoresis and then northern blot analyzed. The biotinylated probe against *mboIIM2* internal sequence, and streptavidin-HRP and ECL chemiluminescent system detection were used. Positions of the full length (FL) and truncated (Trn) transcripts are marked. Bottom panel, ethidium bromide-stained 16S rRNAs were used as loading control. Lane 7, total RNA from non-*mboIIM2* cells

It is reasonable to hypothesize that appearance of a premature TAA stop codon in transcripts produced by *E. coli* RNAP during expression of *mboIIM2ΔA356* frameshifting mutant gene, would trigger ribosome stop followed by transcription and translation uncoupling [55–57]. Dramatic drop in *mboIIM2ΔA356* expression (Fig. 1a) could result from expanding gap between polymerase and the leading ribosome followed by mRNA decay and/or terminator-dependent RNAP dissociation. This idea could indirectly supported by studies on expression of 377- or 378-bp long truncated variants of the *mboIIM2* gene which both relieved polarity effect (Fig. 2a, Additional file 1: Figure S2). In those cases we observed approximately eight- to tenfold increase in the production of M2.MboII short-variant for both *mboIIM2Δ378* and *mboIIM2ΔA356Δ377* genes when compared to the full-length single deletion mutant gene (Fig. 2a, lane 4). In contrast to the host polymerase, T7 RNAP generated expression has been shown to manifest intrinsic resistance to the transcriptional polarity in the absence of T7-specific terminators [58–61]. Since expression of the *mboIIM2ΔA356* could only be 2.3-fold increase by activating λ phage N/*nutL* antitermination system (Fig. 2a, lane 5) it is very likely, that N protein served here as a general elongation factor rather than as antiterminator of any intrinsic terminators [62].

To further investigate the polarity effect as a likely factor of observable expression differences, real time (RT) qPCR technique was used to determine the stability of WT and mutant *mboIIM2* mRNA transcripts synthesized by the host RNAP. Values of the threshold cycle (C_t_) obtained for the particular sections of specific mRNA normalized to external control *cysG* gene [47] reflected relative abundance of the cDNA products located upstream and downstream of the premature TAA stop codon following the ΔA356 frameshift mutation. The total RNA was isolated from strains bearing the native and mutant genes grown under inducing expression conditions (Fig. 2b). We used a set of five primer pairs designed to amplify 98-158 bp fragments of the target cDNA, located at both sides of the premature stop codon (Fig. 2b). We observed a dramatic decrease in the PCR product level generated from the downstream part of the gene in the case of deletion mutant variant *mboIIM2ΔA356*, but not in the wild-type (Fig. 2b).

Next, RT-qPCR assay was performed to compare production level of *mboIIM2ΔA356* and the wild-type mRNA by activating of λ phage N/*nutL* antitermination system (Fig. 2c). Several segments of mRNAs were analyzed, and we observed a two- to threefold improvement in detection of almost all products in case of WT mRNA, while in case of mutated gene the same refers only to the terminal part of the transcript molecule. However, experiments with a Rho-specific inhibitor, bicyclomycin [63] used at concentrations ranging from 5 to 100 μg ml^−1^ showed that production of the full-length M2.MboII protein from *mboIIM2ΔA356* was not affected (Additional file 1: Figure S3). Similarly, no positive effect in production of the full-length M2.MboII was observed when *E. coli* GJ6509 Rho^−^ strain [64] was used as the expression host (data not shown). In contrast, yield of the full-length mRNA increased ca. 30% at the expense of truncated transcript fraction of *mboIIM2ΔA356* in CH1828 *rne*-1 cells with conditionally inactivated RNase E (Fig. 2d, lane 3 vs. lane 4) [65]. Obviously, the uncovered mRNA fragment between ribosome and polymerase may become the target for endoribonucleic digestion. Consistently, full-length transcripts from the wild-type gene are stable in both RNase E phenotypes (Fig. 2d, lanes 1 and 2).

### Determination of the minimal sequence requirements for transcriptional slippage

**Fig. 3:**
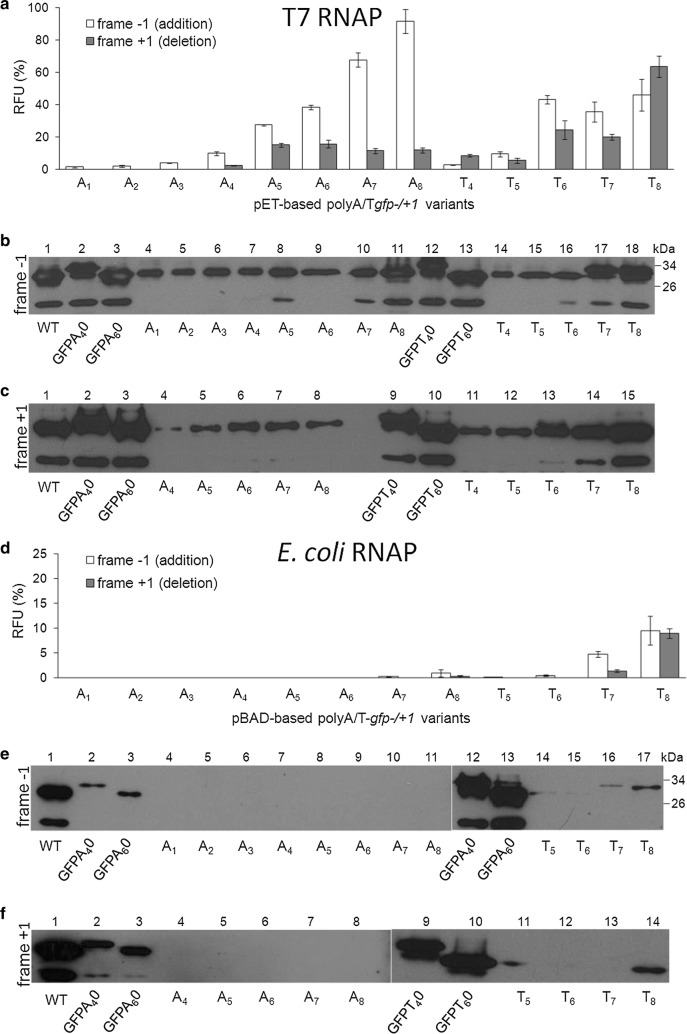
Determination of the poly(A/T) minimal length requirements for transcriptional slippage. Relative fluorescence level of the rescued hybrid GFPs from the particular indel mutant *gfp* genes (Additional file 1: Table S3) measured after 1 h induction with 1 mM IPTG (**a**) or 0.1% l-arabinose (**d**). In-frame *gfpA*_*4*_*0*, *gfpA*_*6*_*0*, *gfpT*_*4*_*0* and *gfpT*_*6*_*0* fusions were used as positive controls and were taken as 100%. Error bars represent standard deviation from at least five independent determinations. Western blotting of the total extracts corresponding to the pET-based *gfp* genes in − 1 frame (**b**) and + 1 frame (**c**). Western blotting of the total extracts corresponding to the pBAD-based *gfp* genes in − 1 frame (**e**) and + 1 frame (**f**)

To determine how a length and kind of nucleotide forming the homopolymeric sequence affect the slippage event, we constructed set of poly(A) and poly(T) vectors with the *gfp* reporter [42] for independent study of expression under the control of the arabinose and T7-phage promoters (Fig. 3, Additional file 1: Table S3). Each tested fusion gene contained slippery sequence attached upstream to the out-of-frame *gfp* reporter (− 1 or + 1). These fusions did not appear to have an intracistronic transcription polarity effect and enabled direct measurement of the transcriptional slippage ability [52]. All nucleotide sequence changes were created to obtain variants of runs with successively increased number of A or T nucleotides (Additional file 1: Table S3).

Bacterial cultures with plasmids carrying out-of-frame *gfp* reporters were induced with either 1 mM IPTG (pET-derivatives with T7 promoter) or 0.1% (6.7 mM) l-arabinose (pBAD-derivatives with P_araBAD_ promoter), for 1 h at 37 °C. The concentration of the arabinose inductor was chosen to balance the sevenfold faster transcriptional rate of T7 RNAP [66]. Each time the obtained fluorescence levels of parallel cell lineages were referred to appropriate in-frame controls on either pET24a or pBAD24 backbone, and containing the same number of natural amino acids residues [52]. Our results (Fig. 3a–d) indicate strong ability of T7 RNAP to rescue poly(A) and poly(T) frameshifted *gfp*-*1* gene variants by nucleotide insertion, retained up to 90% and 50% of the in-frame control expression levels, respectively (Fig. 3a), while barely none fluorescence was detected in case of the host polymerase, with only 5% and 10% for poly(T_7_/_8_), with an opposite preference to the T7 enzyme (Fig. 3d). Remarkably, we show that T7 RNAP is potent to slip on such short homopolymers as three As and four Ts in a raw. In parallel, no detectable activity was identified for the host RNAP for homopolymers shorter than seven Ts and eight As, respectively (Fig. 3d).

Moreover, restoration of *gfp*+*1* by T7 RNAP through deletion was significantly low (Fig. 3a), especially in case of poly(A)s where the efficiency of such an event never reached 20%. In contrast, restoration by poly(T) nucleotides was at threefold higher efficiency, reaching over 60% in case of poly(T_8_). In parallel, *E. coli* RNAP revealed higher preferences for poly(T) erroneous deletions, giving 10% efficiency for poly(T_8_) sequences and none for the poly(A) examined runs (Fig. 3d). Also, the fluorescence microscopy preparations of cells carrying the *gfp(A/T)*-*1* fusions presented in Additional file 1: Figure S4 were consistent with the results of quantitative measurements. Generally, the longer the homopolymer length, the higher the level of rescue by insertion was observed. In contrast, for deletion slippage only poly(T) series apply to this rule.

All obtained results well correlated with immunodetection of the GFP hybrid protein levels from ER2566 and DH10B cell lysates (Fig. 3b, c, e, f, respectively).

### NGS mRNA analysis confirmed nucleotide bias of the host and T7 phage RNAPs in slippage induction

**Fig. 4:**
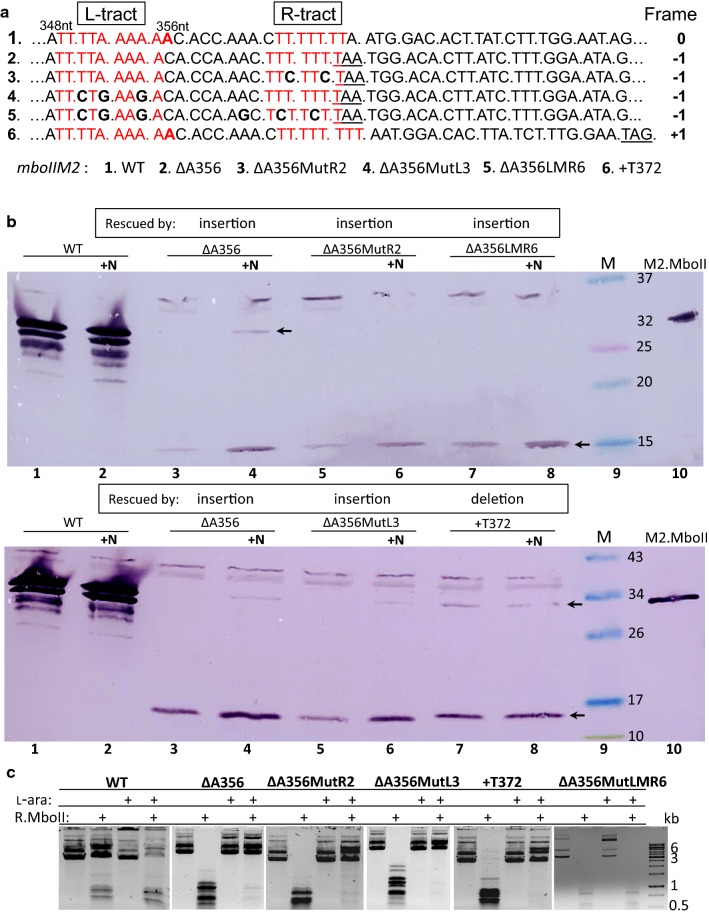
Effect of N/*nutL* antitermination on expression of A/T homopolymers potent to bypass A356 deletion mutation of the *mboIIM2ΔA356* gene. **a** Details of sequence of the selected indel mutant genes (short names). Premature stop codons are underlined. T/A homopolymer runs are marked in red. Substituted nucleotides and the position of the A356 nucleotide are in bold. **b** Western blot of expressed gene mutants in DH10B strain in the absence or presence (+ N) of λ phage N/*nutL* antitermination co-expression (both 0.1% l-arabinose induced for 1 h at 37 °C). Top and bottom panels: Equal amounts of total protein extracts from cultures harboring appropriate gene mutants were analyzed after 12.5% SDS-PAGE and immunodetection with anti-M2.MboII antibodies. Lanes 9, molecular size markers (BioRad or Fermentas, respectively), lanes 10, M2.MboII purified protein. Bands of full-length (32 kDa) and short variant of M2.MboII (14.5 kDa) are pointed by arrows. **c** The relative protection level of the MboII-specific sites against R.MboII digestion in plasmids bearing the corresponding *mboIIM2* indel mutants

Next, we tested whether observable differences in nucleotide type preferences are indeed significant. Recently NGS analysis of transcript indel polymorphism covering 375 nt proximal part of *mboIIM2ΔA356* gene synthesized by the T7 RNAP was performed [16]. Now, to evaluate the extent of erroneous transcription led by *E. coli* RNAP analogous procedure was done. Comparative analysis showed four most dominant slippage sites, the same in case of both RNAPs (Additional file 1: Figure S5). However, the relative values of indel frequency presented variable amplitude of the slippage errors. In general, *E. coli* RNAP contributes to 8 times lower overall frequency of the nucleotide insertion and deletion (0.0066% and 0.007%, respectively) (Additional file 1: Figure S5, Additional file 1: Table S4). Insertion slippage level in the corresponding homopolymer runs was the highest for T_6_ (13 nt) and T_7_ (364 nt) tracts (0.82% and 0.75%, respectively) and lower for A_7_ (279 nt) (0.39%). Apparently, poly(T)-type homopolymers induced higher insertion frequencies which correlates well with results obtained for expression of *gfp* fusion mutants (Fig. 4). The most potent region for insertion generation by the T7 RNAP—poly(A_5_) (351–355 nt), barely induces slippage in the case of the host RNAP (10.45% vs. 0.062%). Furthermore, the poly(T_7_) run (364–371 nt) appeared to be roughly half as efficient (0.75 vs 1.19%, respectively). The combined frequency of insertions in both poly(A) and poly(T) regions, the key prerequisite of the A356 deletion mutation rescue of *mboIIM2ΔA356*, is 14 times lower for the host RNAP than in case of the T7 enzyme (Additional file 1: Figure S5). Interestingly however, we did not notice any significant role in slippage induction for the T_5_ tract located between 314 and 318 nt in both cases. It strongly suggests that it is not only the length of a homopolymer run, but also the context of the adjacent nucleotide sequence that is important for the incidence of transcriptional slippage [16, 26, 67].

In order to evaluate the nucleotide-type preference/bias of *E. coli* RNAP for slippage in vivo, the *mboIIM2ΔA356* single deletion mutant gene with several changes in the homopolymer tracts important for rescue, were used (Fig. 4a). The site-specific nucleotide replacements systematically interrupted the continuity of the homopolymeric A_5_ run (l-tract, 351–355 nt), and the R-tract, consisting of seven T (364–370 nt), upstream of the premature TAA stop codon in the gene (Fig. 4a, [16]). To better visualize the expression results we enhanced this process by λ N stimulation (Additional file 1: Figure S6). The ΔA356MutR2 mutant gene, with interrupted poly(T_7_) sequence, resulted in a significantly decreased full-length methyltransferase production when compared to the ΔA356MutL3 mutant with interrupted poly(A_5_) run (visible full-length protein production, Fig. 4b, top panel, lanes 5 and 6 vs. bottom panel, lanes 5 and 6). These results testify that A356 deletion mutation was bypassed mainly by U rather than A insertion and are in good agreement with indel mRNA polymorphism pattern for *E. coli* RNAP (Additional file 1: Figure S5) and, which is opposite to the T7 RNAP action [16]. Since no slippery sequences in this region remained in the ΔA356LMR6 mutant gene, no production of the full-length protein was found and consequently no MboII-specific methylation of DNA was detected (Fig. 4c). Western blot analysis of the protein extracts from cells expressing *mboIIM2* indel variant genes indicated no reduction, but instead two- to threefold stimulatory effect of N protein on the production of both M2.MboII protein forms was observed (Fig. 4b). As reported previously, N-dependent inhibition of the host RNAP slippage preferentially affects nucleotide deletions [62]. We confirmed this in case of the + 1 frameshift mutant + T372 (T_8_, 364–372 bp) expression. While in-frame short product synthesis was maintained on the same level under both conditions (N^+^/N^−^), the full-length rescue product quantity was significantly reduced (40%) in the presence of N (Fig. 4b, bottom panel, lane 8).

### Identity of the upstream and downstream bases adjacent to the slippery sequence impacts transcription slippage efficiency

To study the influence of nucleotide type on induction of slippage, several *gfp*-*1* and *gfp *+ *1* reporter genes were constructed (Additional file 1: Figure S7) varying in two nucleotides located directly upstream to the slippery-prone sequences: *gfpA*_*5*_ and *gfpT*_*5*_, respectively. Results for the tested di-nucleotides pairs were normalized to appropriate in-frame controls [52] and compared to the ones obtained for T_2_A_5_∓1 and A_2_T_5_∓1, respectively. In the case of A_2_T_5 _− 1 template sequence, replacing the 5′-AA with CA, AC and GG did not affect slippage, while we observed 25% increase when AG and CC pairs were analyzed. Threefold decrease was shown in case of deletion slippage of C_2_T_5_ + 1. In contrast, changing the primary TT of the T_2_A_5_-1 motif to TC and CC resulted in 35% and 75% decrease in insertion-mode slippage, respectively. CT and GG substitutions gave no effect, and deletion slippage decreased by ca. 50% in case of CC pair. The fluorescence results were confirmed by immunoblotting of the GFP hybrid (Additional file 1: Figure S7c).

To study the effect of downstream located nucleotide, the primary C in T_2_A_5_C − 1 and A_2_T_5_C − 1 was replaced with G, T or A, respectively (Additional file 1: Figure S8). Results showed only a moderate impact of the n + 1 nucleotide. In case of T_2_A_5_ − 1 series 2.2- and 2.7-fold decrease of insertion slippage was observed for G and T alterations. In contrast C→A substitution in A_2_T_5_C − 1 did not result in any change and 50% increase was observed in case of C→G.

In both cases the poly(A_5_) homopolymers were more liable to the upstream and downstream nucleotide base changes.

## Discussion

This study aimed for systematic investigation of transcriptional T7 phage and *E. coli* RNAPs slippage potential. Three parameters were analyzed: homopolymer length, template type, and frameshift direction preference. By using a set of out-of-frame *gfp* reporter fusion genes we were able to monitor restoration of the native frame by a predominant single-nucleotide insertion or deletion events in upstream located A/T mononucleotide tracts. We show that the minimum homopolymer length requirement for the productive in vivo transcriptional slippage by *E. coli* RNAP is 7 T_s_ in a row (Fig. 3). In contrast, induction of effective slippage for T7 RNAP was possible with nucleotide tracts as short as either three As or four Ts (Fig. 3). Likewise, a remarkably high bypass of the frameshift mutations located in the proximal part of the *gfp* gene was described in *Bacillus subtilis* cells [6]. Little inclination of the *E. coli* enzyme to slip is in agreement with earlier reports concerning the expression of *lacZ* reporter fusions [37, 62, 68, 69] and transcriptome-wide spectrum of indels analysis [40]. Accordingly, it was shown that detectable effect of indel mutation restoration required more than 7 As or Ts in a row. Our studies demonstrated a different slippage frequency pattern for the A/T homopolymer for phage and host RNAPs. Whereas an insertion slippage by T7 RNAP occurs preferentially at the poly(A) than poly(T) runs, strong bias for T nucleotides for the host RNAP is observed. At first glance, it is not surprising and could be explained by differences in the rU:dA structure stability (exceptionally unstable) when compared to the rA:dT duplexes [37, 70–72]. However, other causes may also rely upon various properties of the T7 phage RNAP, representing a single subunit class of enzymes, and multisubunit bacterial RNAP [73], even just because there are differences in the auxiliary factor dependency [74–77]. Recently reported interesting results shed some light on the complexity of this issue. As was shown, replacement of the Us tract with As in the Tφ terminator allowed to reach half of the maximum termination efficient by T7 RNAP in vitro [26], which indicates its alternate transcriptional signals tolerance. Moreover, our study of indel frequency by NGS transcripts composition analysis of the *mboIIM2* gene showed threefold increase of insertion type slippage over deletion, based on data from four active homopolymeric runs performed by the host RNAP, and its 6.5 times lower overall slippage effectiveness in comparison with the T7 enzyme (Fig. 4). It was further verified by in vivo experiments with T7 RNAP which revealed that backward slippage generated nucleotide insertion occurs 4–6 times more often than by deletion in the 5–8 nt long runs (Fig. 3). This is in agreement with earlier suggestions that preference for transcriptional backward slippage is greater than forward slippage, at least in case of T7 RNAP [78]. What is more, insertion events in out-of-frame *lacZ* mutants with a range between 7 and 11 adenine stretches were at least twice as much efficient as slippage by deletion [62]. We are aware that our results are just an estimate, not an absolute calculation, since as was shown elsewhere, in long homopolymers the slippage potential is more frequently divided into a “forward” and “backward” event differentiation [16, 37, 62]. In fact, slippage by enterobacterial RNAP was shown to occur in a run of 9 As or Ts and longer with variable directionality [35, 37, 68, 79, 80]. Both frameshifting types were detectable in a semi-synthetic 3A′ reporter gene containing T_9_ slippery sequence, with increased forward (base insertion) over backward frameshifting, in a growth phase dependent manner [81]. Likewise, a T_9_-run in the template strand of *dnaX* gene mRNA sequence of *Thermus thermophilus* was found to induce both − 1 and + 1 frameshift in *E. coli*, with predominance of multiple insertions [30, 82]. Similar observation concerns *E. coli lacZ* gene (A_11_) [37], *spa13* (A_10_) and *mxiE* (T_9_) genes of *Shigella flexnerii* [80, 83], and the *mur* gene family (A_10_) of *Buchnera aphidicola* [35] and also *tssM of Citrobacter rodentium* [79]. Interestingly, yeast RNAP II and *E. coli* RNAP exhibited the opposite slippage directionality in the A_11_-tract, weak insertion propensity and deletion predominance, respectively, in an experiment performed under the same transcription in vitro conditions [84]. All of the above examples suggested variable and vicinity context-dependent ability of RNAP to make indel errors [68]. In fact, we have shown that identity of nucleotides upstream and downstream of the slippage-prone sequence could moderately change the slippage efficiency, ranging from 25% to almost 1.5-fold (Additional file 1: Figures S7 and S8). This is likely related to the fact that the rate of misincorporation by misalignment is significantly increased when more than one base pair downstream of the RNAP active site in DNA duplex is melted [85]. To some extent it can be suppressed by stabilizing action of λ N antitermination protein [62, 86]. Obviously, we cannot exclude the involvement of much broader nucleotide context of upstream sequences that could form a secondary structure which could negatively/positively impact slippage. For instance, Penno and collaborators [11] showed realignment in two directions at the stem-loop RNA, stimulating slippage at T_6_–T_9_ runs with predominance of the base deletion events and several-fold decrease in slippage by replacement of the penta-nucleotide segment in the upstream sequence [68, 80].

Although the two examined enzymes represent quite different classes of polymerases, the length of the RNA:DNA hybrid and size of transcriptional bubble are conserved between single and multisubunit RNAPs and undergo intrinsic endonucleolytic regulation, at least in case of the last one [87]. A stable 8–10 bp RNA–DNA hybrid in the transcription elongation complex is essential for keeping the RNA 3′terminus engaged with active site of *E. coli* RNAP in register with the template strand [88, 89]. Protein-nucleic acid interactions upstream and downstream of the hybrid region play primary role in holding together all components of the polymerase elongation complex [89, 90]. As was shown, increased slippage phenotype in some *rpoB* mutants that cluster along the RNA strand of the hybrid, involved transient separation of the RNA and DNA template strand in the elongation complex and resulted in the loss of transcription register [69, 84, 91]. However, tight contact of the RNAP with RNA:DNA hybrid is limited to the first 2–3 3′-proximal bp of the heteroduplex whose base pairs complementarity is sensed and recognized [92, 93]. Destabilization in this part of the RNA:DNA hybrid triggers realignment and promotes slippage.

Besides that, it is also reasonable to consider observed differences in the light of proofreading process involvement and participation of the auxiliary factors which determine non-uniform mechanism of fidelity among the host and phage RNAPs [94]. Importantly, T7 RNAP neither exhibits intrinsic proofreading activity [95] nor mRNA elongation process involves transcriptional proteins, in contrast to the host polymerase [5, 74–77]. This is especially evident in the case of the *mboIIM2ΔA356* single deletion mutant gene expression. We demonstrated a 40 times greater difference in ratio of ΔA356 mutant corrective slippage, as well as overall expression of the in-frame part of the gene in favor of the T7 RNAP (Fig. 1). That result was in contrast to *gfp* fusions with the proximal located out-of-frame segments. Such discrepancy in effectiveness is due to strong transcriptional polarity which the host RNAP transcription process was subjected to. Tight coupling between leading ribosome and RNAP plays a central role in controlling gene expression, mainly by preventing nascent terminator hairpin formation and binding of the transcription termination Rho global regulator [96]. Moreover, mRNA translating ribosomes modulate formation of nascent RNA structure [97], block RNAP backtracking and protect mRNA from RNase degradation [65, 66, 98]. Uncoupling between transcription and translation causes RNAP to adopt at non-productive state [55], as well as elongation complex disruption and dissociation within coding sequences [96, 99, 100]. Consequently, accelerated mRNA decay [101] and degradation of truncated polypeptide take place [102, 103]. Here, we showed greater instability of the *mboIIM2ΔA356* full-length mRNA transcript (Fig. 2d) as a result of premature translation termination. Comparative analysis of the *mboIIM2* transcript segments by real-time RT-qPCR confirmed its terminal part instability (Fig. 2b). Since active translation can protect the mRNA against an attack of cellular endoribonucleases, we investigated the role of RNaseE in *mboIIM2ΔA356* mRNA degradation using *E. coli* CH1828 strain carrying the *rne*-*1* thermosensitive mutation [65]. RNase E is known to initiate the decay of most individual mRNAs by internal cleavage in the single-stranded untranslated region rich in AU residues [104]. We showed that expression of the *mboIIM2ΔA356* gene under RNase E deficient conditions significantly reduced degradation of the full-length transcript (Fig. 2d). N/*nutL* antitermination system, also known as a slippage inhibitor in the very long A/T tracts [62], only partially suppressed the observed transcription polarity effect (Fig. 2c). However, deletion of the fragment of gene upstream of the A356 frameshifting site almost completely abolished it (Fig. 2a). We showed that N did not act as an actual slippage reducer in this case, but rather as a factor stimulating elongation rate of RNAP by pause elimination [62, 105, 106]. Further experiments showed positive stimulation of transcription in both frames (− 11/+ 1), when A/T homopolymers were shorter than 8 nucleotides (Fig. 4). An earlier study had shown [62] that N served more significantly as an inhibitor of slippage which occurred via nucleotide deletion. We found this to be the case for the + T372 insertion mutant *mboIIM2* gene with eight consecutive T (364–372 bp) (Fig. 4b, bottom panel). Interestingly, in the absence of the *nutL* sequence, the N protein inhibits overall expression process in both, the native and deletion mutant gene (Additional file 1: Figure S6a, lanes 5 and 11), consistent with the effects described elsewhere [57, 107–109]. Since the presence of GreA and GreB auxiliary factors increases fidelity, their contribution to the slippage process cannot be excluded. This idea was supported earlier by a reporter assay in Δ*greAB* cells where shift-prone A_9_ tract within *lacI* gene resulted in a several dozen times increased frameshift frequency over wild-type, however, transcription errors were not measured directly [5, 33]. In fact, to induce slippage, *E. coli* RNAP requires very long stretches of monotonous A/T tracts which are rather rarely found in the chromosome [110]. Especially, one might assume that the mechanism of indel error formation by flipping-out of a base one strand of the nascent RNA:DNA hybrid gives different results than simple nucleotide misincorporation, because the nucleotide change is quantitative and no qualitative. Misincorporation does not allow to form the correct base pairing between improper NTP with the template DNA, and such distortion impairs further mRNA extension and the enzyme translocation. Since homopolymeric indel nucleotide is always cognate and matched to the *i *+ 1 site of the template strand, it presumably neither perturbs the 3′end of the hybrid stability nor enforces backtracked pauses of the enzyme. Generated errors can possibly be corrected by slow intrinsic proofreading rather than stimulating action of Gre/DskA factors, either in enhancement of the cleavage activity or in restart of the backtracked RNAP [111]; Roghanian et al. [75]; Satory et al. [76, 112]. For this reason, GreAB action might have a rather limited impact on the slippage process. Indeed, we tested expression level of the *gfpT*_*8*_-*1* variant generated by *E. coli* host RNAP in the WT as well as in its isogenic Δ*greAB* strain and we did not find an increase in the rescue of GFP production level (Additional file 1: Figure S9). We could not test the polyA homopolymeric sequence due to slippage occurring below the level of detection. Recently, several reports did not confirm significant effects on transcriptional indel rates as a result of the absence of Gre factors, with the exception of preponderance of G→A misincorporation in mutants lacking GreA [111–114].

In conclusion, RNAP propensity to slippage involves trade-offs between accuracy, speed and processivity of transcription. Viral T7 RNAP manifests far greater inclinations to the transcriptional slippage than *E. coli* RNAP. This phenotypic variability possibly plays an important role in driving bacteriophage adaptation and therefore could be considered as beneficial in that case. However, from biotechnological and experimental point of view it creates some problems. High level of frameshift errors in nascent RNA results in expression and accumulation of misfunctioning trunckated proteins. This could be detrimental for global cell physiology by metabolic burden and necessity to launch energy consuming “cleansing” mechanisms as a response to proteotoxic stress, as well as loss of control over the quality of protein preparations. Taking all above into consideration, this strongly argues for employing bacterial expression systems stocked with proofreading mechanism as a recommended solution, especially when expression profile of an indel mutated gene must be unambiguously investigated. However, high poly(A)-based backward slippage propensity of T7 RNAP can be utilized in case of two or more combined genes expression as a new possibility for transcription down-regulation of a selective indel gene, to obtain desired protein amounts.

## Additional file

**Additional file 1.** Additional Tables S1–S4, Figures S1–S9.
