# Supplementary material for: Non-programmed transcriptional frameshifting is common and highly RNA polymerase type-dependent
Source: Microb Cell Fact. 2018 Nov 24;17:184. doi: 10.1186/s12934-018-1034-4 (PMC6260861; doi:10.1186/s12934-018-1034-4)
Supplement: Supplementary file 1 — Additional file 1. Additional Tables S1–S4, Figures S1–S9. [file 12934_2018_1034_MOESM1_ESM.pdf]

Additional file1: Figures S1-S9, Tables S1-S4

Non-programmed transcriptional frameshifting is common and highly RNA polymerase type-dependent

Koscielniak D., Wons E., Wilkowska K. and Sektas M.*

Department of Microbiology, University of Gdansk, Wita Stwosza 59, 80-308 Gdansk, Poland

*To whom correspondence should be addressed: Tel: (+4858) 5236068; Fax: (+4858) 5236073; Email: marian.sektas@biol.ug.edu.pl

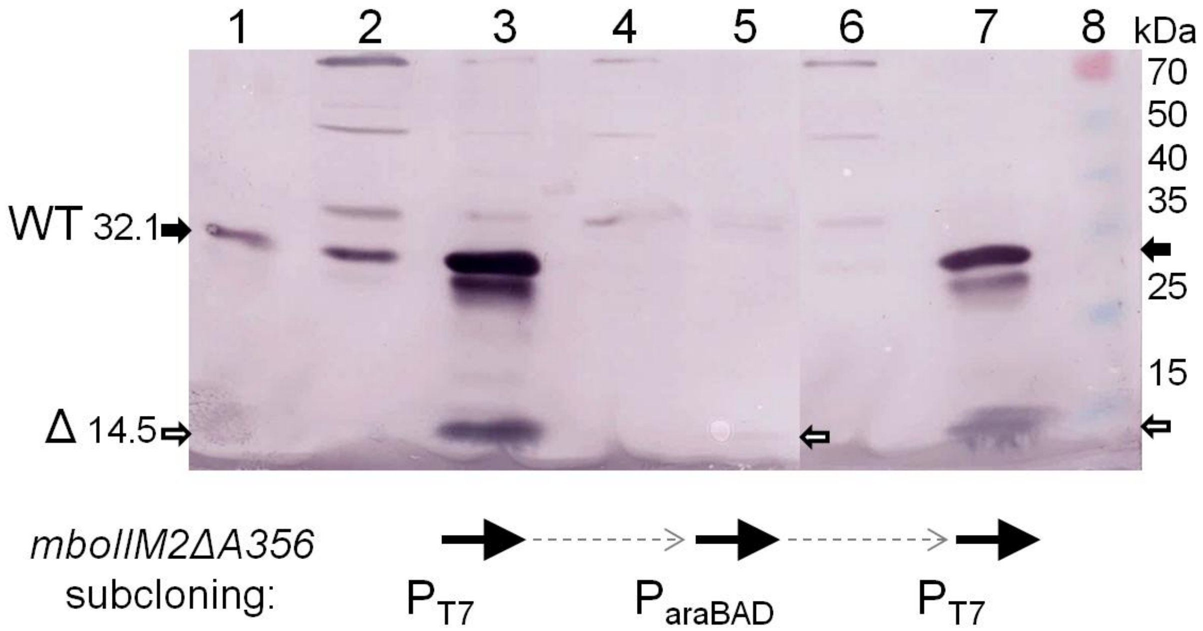

Figure S1. T7 RNAP slippage-dependent rescue of nucleotide deletion mutation in the *mboIIM2ΔA356* gene by western blotting. Immunodetection of M2.MboII protein variants with anti-M2.MboII serum and BCIP/NBT color development system. Lane 1, purified M2.MboII protein, lanes 2 and 3, lysates from non-induced and 1 mM IPTG induced *E. coli* ER2566 cells carrying pETmboIIMB.4 plasmid (*mboIIM2ΔA356*); lanes 4 and 5, lysates from non-induced and 0.1% L-arabinose induced *E. coli* DH10B cells carrying pBADmboIIMB.4 plasmid (*mboIIM2ΔA356*); lanes 6 and 7, lysates from non-induced and 1 mM IPTG induced ER2566 cells carrying pETremboIIMB.4 plasmid (*mboIIM2ΔA356*). The positions of full-length (WT) and short-form (Δ) of M2.MboII protein are indicated by arrows.

mboIIM2Δ378

(349 nt) (378 nt)

…**TTA AAA AAC ACC AAA CTT TTT TTA ATG GAT** *CCG AAT TCG AGC TCC GTC GAC AAG CTT GCG GCC GCA CTC GAG CAC CAC CAC CAC CAC CAC* **TGA…**.

STOP

mboIIM2ΔA356Δ377

(349 nt) (377 nt)

…**TTA AAA ACA CCA AAC TTT TTT TAA TGGAT***CCGAATTCG*….

STOP

Figure S2. Details of the DNA sequence of the two *mboIIM2* deletion mutant genes used in experiment presented in the Fig. 2. The part of the wild-type *mboIIM2* sequence is marked in bold; the part of the pET24a vector sequence is italicized. In red, BamHI restriction endonuclease recognition sequence. Note, that molecular mass of resulting protein from *mboIIM2Δ378* and *mboIIM2ΔA356Δ377* is 17,1 and 14.5 kDa, respectively.

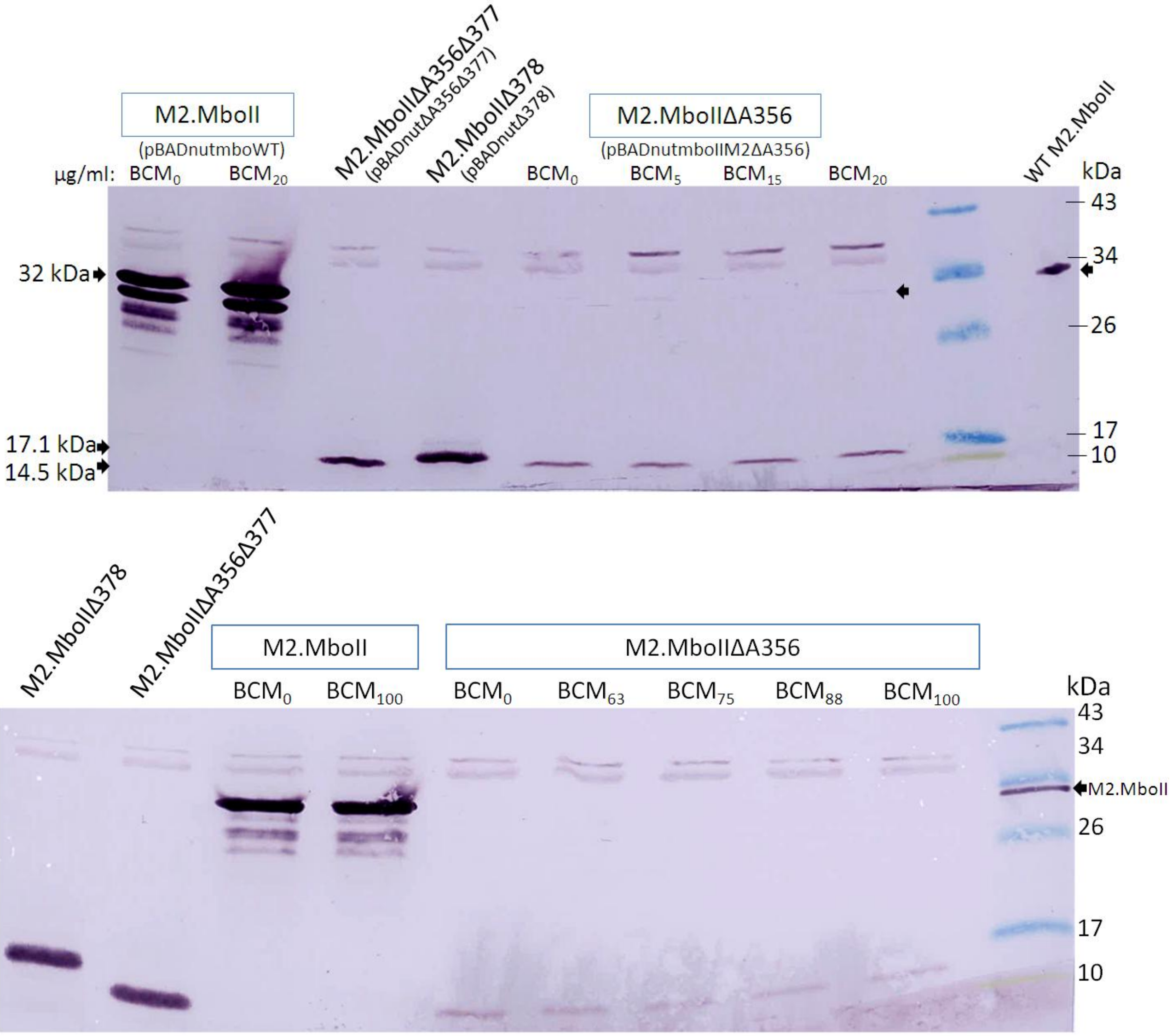

Figure S3. Bicyclomycin (BCM) effect on intracistonic transcription polarity during *mboIIM2ΔA356* expression. Western blotting detection of the full-length and short M2.MboII products isolated from DH10B carrying *mboIIM2ΔA356* gene on pBADnutmboIIM2ΔA356 plasmid. BCM treatment of cells at concentration range 0-20 μg/ml (top panel) and up to 100 μg/ml (bottom panel) during induction by 0.1% L-arabiniose for 1 hour at 37°C. Molecular size controls: WT M2.MboII (pBADnutmboWT) and its truncated variants M2.MboIIΔ378 (pBADnutΔ378) and M2.MboIIΔA356Δ377 (pBADnutΔA356Δ378) are indicated.

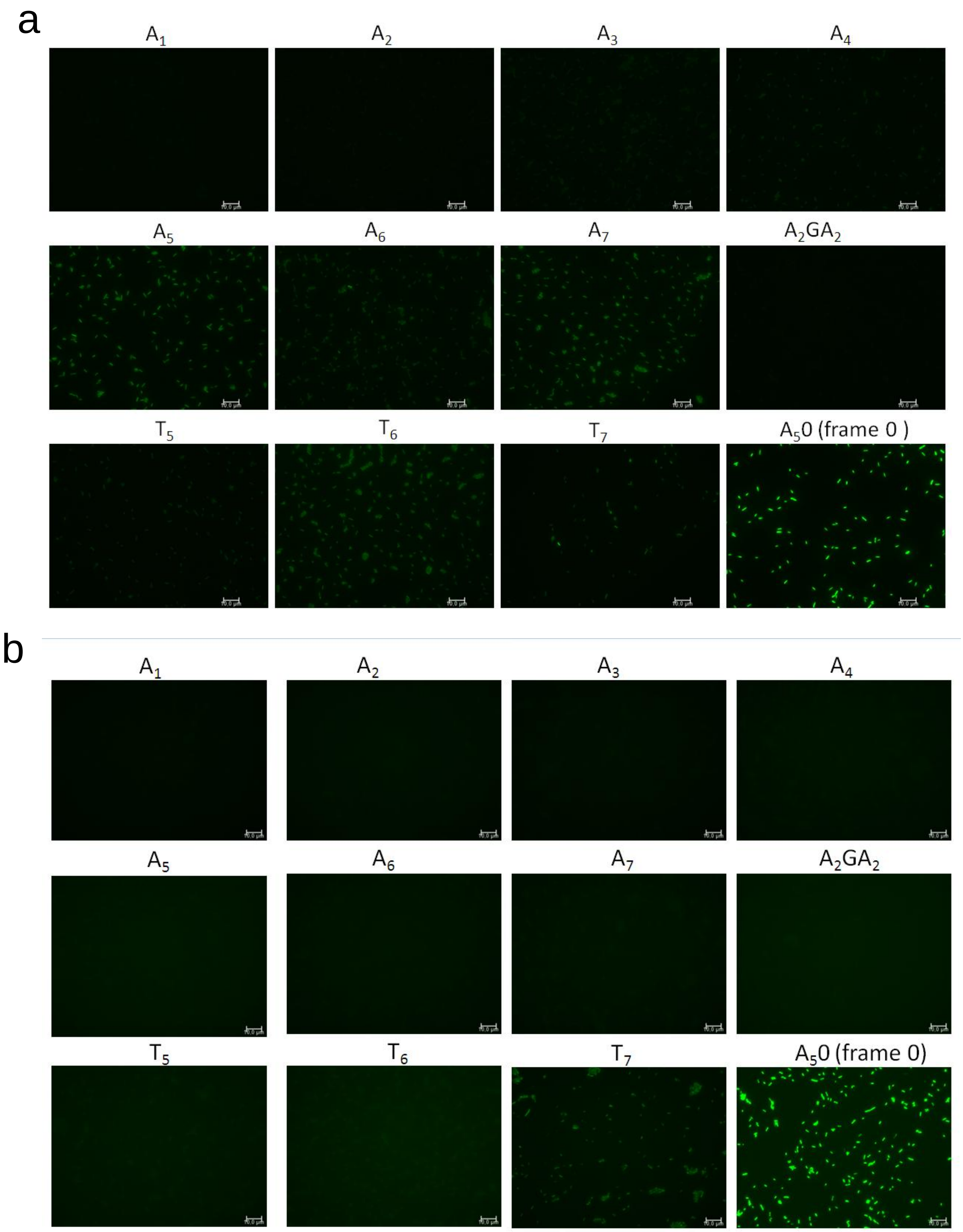

Figure S4. Relative transcriptional slippage efficiency in various *gfp* fusion genes detected by fluorescence microscopy. **a** pETmingfp(A/T)-1 series. **b** pBADmingfp(A/T)-1 series. Scale bar ≈ 10μm.

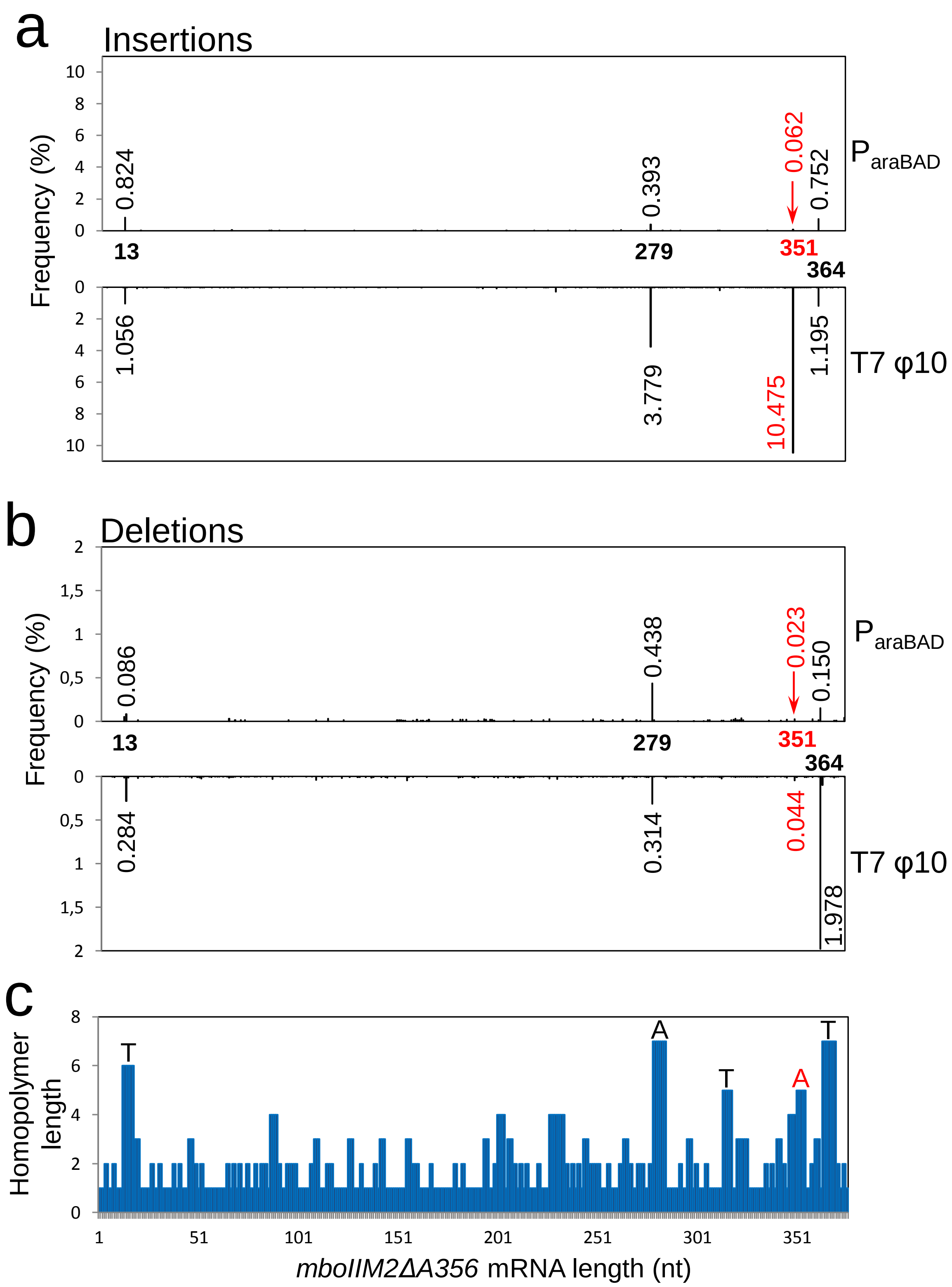

Figure S5. *E. coli* and T7 RNA polymerase-based transcriptional indel error distribution within *mboIIM2A356* mRNA (1-376 nt) analyzed by the NGS method. **a** Distribution and frequency of nucleotide deletions. **b** Distribution and frequency of nucleotide insertions **c** Nucleotide homopolymer distribution. High slippage inducing A_5 site is indicated in red (Wons et al., 2015).

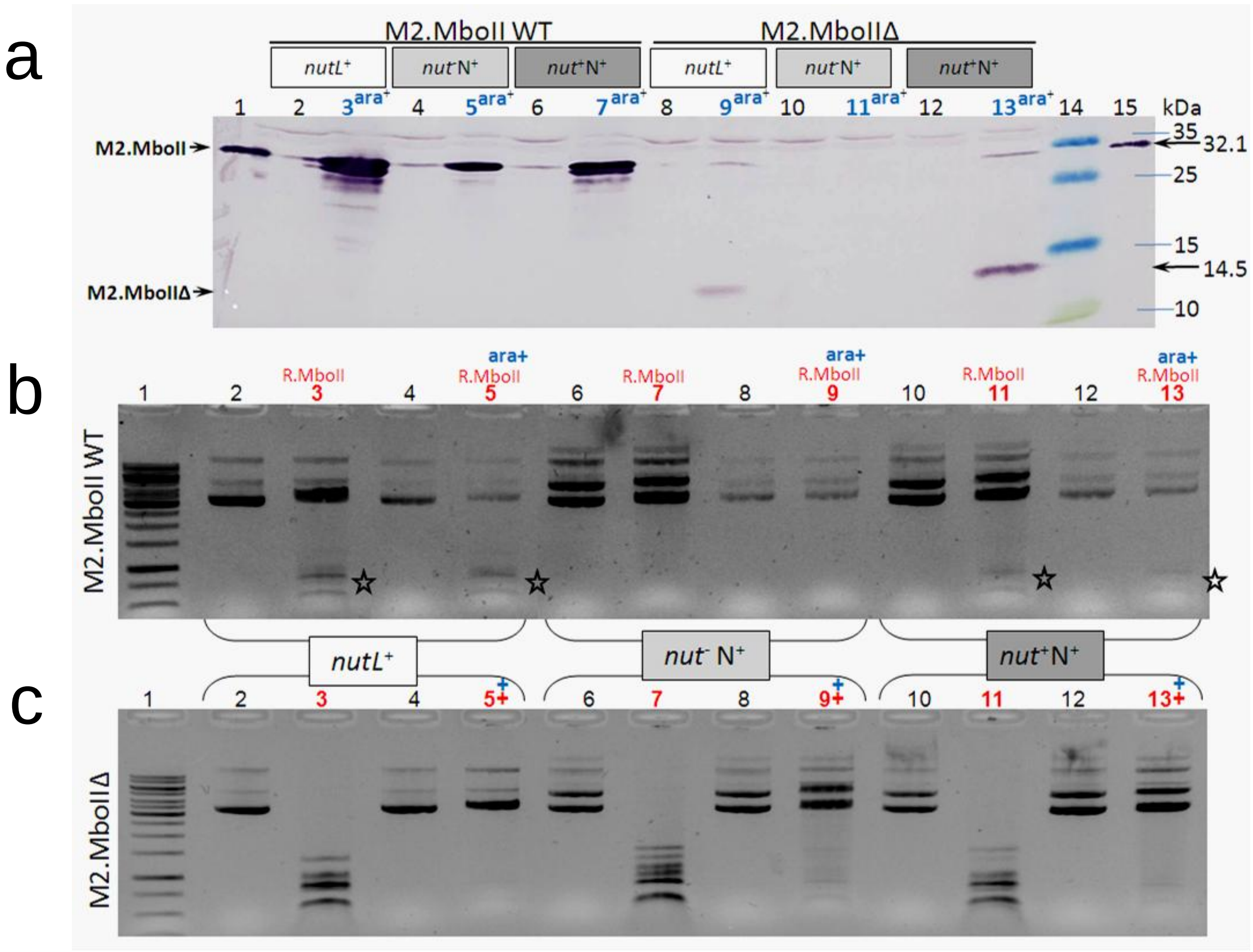

Figure S6. N/*nut*L antitermination enhance expression of indel mutant gene. **a** *nut*L sequence, N protein and N/*nut*L λ phage antitermination system on expression of P_{araBAD} promoter-dependent wild-type (lanes 2 - 7) and single deletion mutant *mboIIM2ΔA356* genes (lanes 8 - 13). Bacterial lysates were run on SDS-12.5% PAGE, western blotted and immunodetected for M2.MboII protein. Lanes 2, 4, 6, 8, 10 and 12, total protein extracts from DH10B non-induced cells; lanes 3, 5, 7, 9, 11 and 13, total protein extracts from 0.1% L-arabinose induced cells (2 h at 37°C). Lanes 1 and 15 – purified M2.MboII protein. Lane 14 – prestained molecular size marker (Fermentas). **b** Relative level of plasmid methylation by M2.MboII produced by the wild-type *mboIIM2* expression, challenged by R.MboII endonuclease digestion. Gene expression not supported by antitermination system (*nut*L$^+$, *nut*L$^-$N$^+$) and supported by λ phage antitermination (N/*nut*L) is shown. Plasmid isolates correspond to cell extracts in lanes 2 - 7 of Panel **a**. Plasmid DNAs (lanes 2, 4, 6, 8, 10 and 12) were digested with R.MboII enzyme (lanes 3, 5, 7, 9, 11 and 13) and resolved on 0.8% agarose gel and visualized with ethidium bromide. Lane 1 – molecular weight marker 1 kb (Fermentas). Inverted image of the gel is shown. **c** Relative level of plasmid methylation by M2.MboII produced by the *mboIIM2ΔA356* mutant gene expression, challenged by R.MboII endonuclease digestion. Gene expression not supported by antitermination system (*nut*L$^+$, *nut*L$^-$N$^+$) and supported by λ phage antitermination (N/*nut*L) is shown. Plasmid isolates correspond to cell extracts from lanes 8 - 13 of Panel **a**. Plasmid DNAs (lanes 2, 4, 6, 8, 10 and 12 – undigested) were digested with the R.MboII enzyme (lanes 3, 5, 7, 9, 11 and 13) and resolved on 0.8% agarose gel electrophoresis. Lane 1 – molecular weight marker 1 kb (Fermentas). Inverted image of the gel is shown. ☆Unprotected fraction of plasmid DNA.

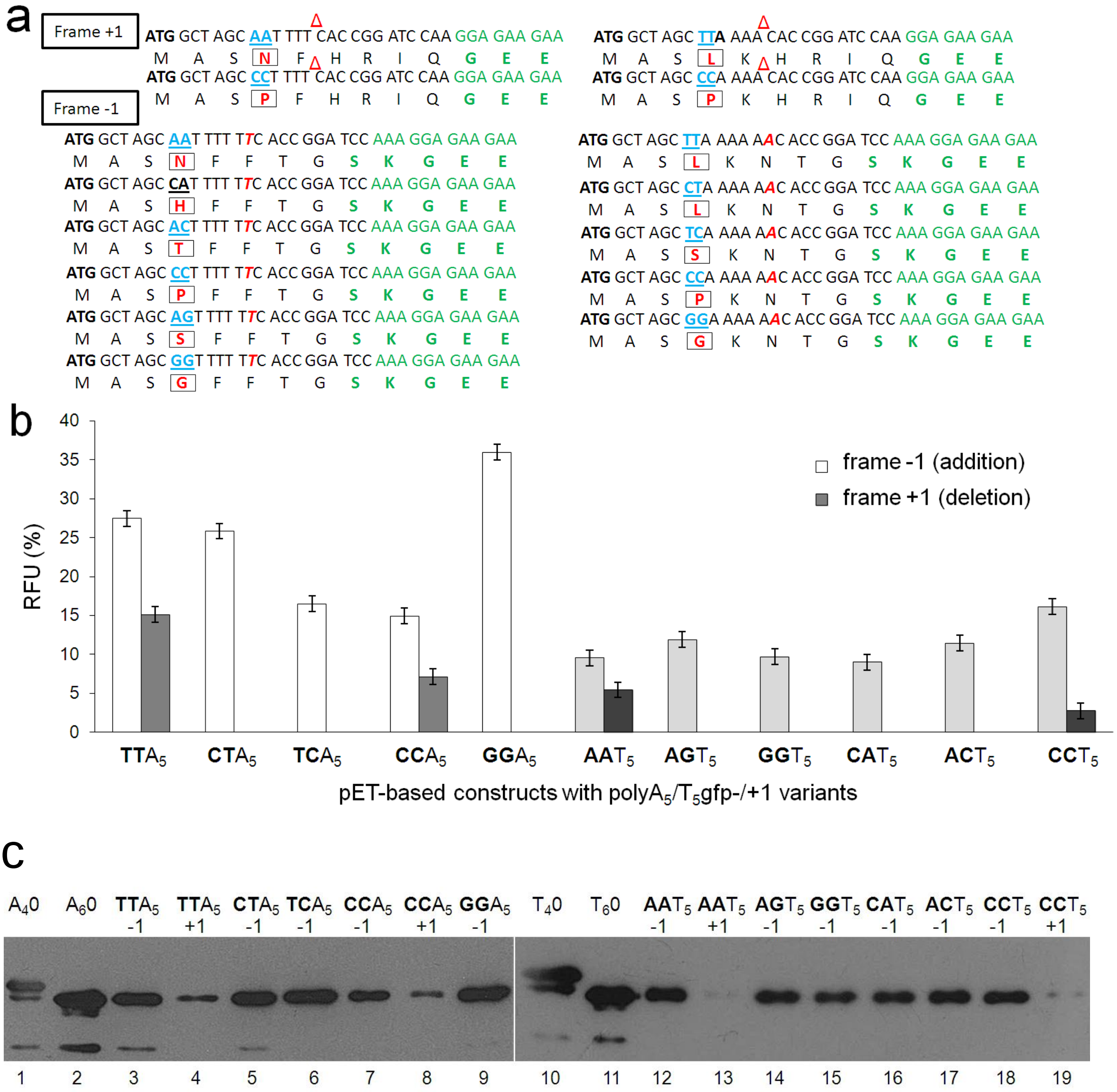

Figure S7. Effect of preceding dinucleotide on forward and backward slippage efficiency of T7 RNAP in poly(A_5/T_5) runs. **a** Details on the DNA and amino acid sequences in particular *gfp* constructs after single nucleotide deletion (frame +1) or addition (frame -1). Dinucleotide substitutions are marked in blue; inserted T/A nucleotides are in red and italicized; Δ – site of the A/T deletion. In green, part of the wild-type *gfp* DNA/amino acid sequence is given. **b** The influence of the nucleotides preceding the poly(A) and poly(T) sequences on slippage efficiency reflected by rescued GFP fluorescence after normalization to appropriate GFP frame 0 controls. Error bars represent standard deviation from at least three independent determinations. **c** Expression level of the corresponding *gfp* constructs assayed by western blotting and immunodetection of GFP hybrids.

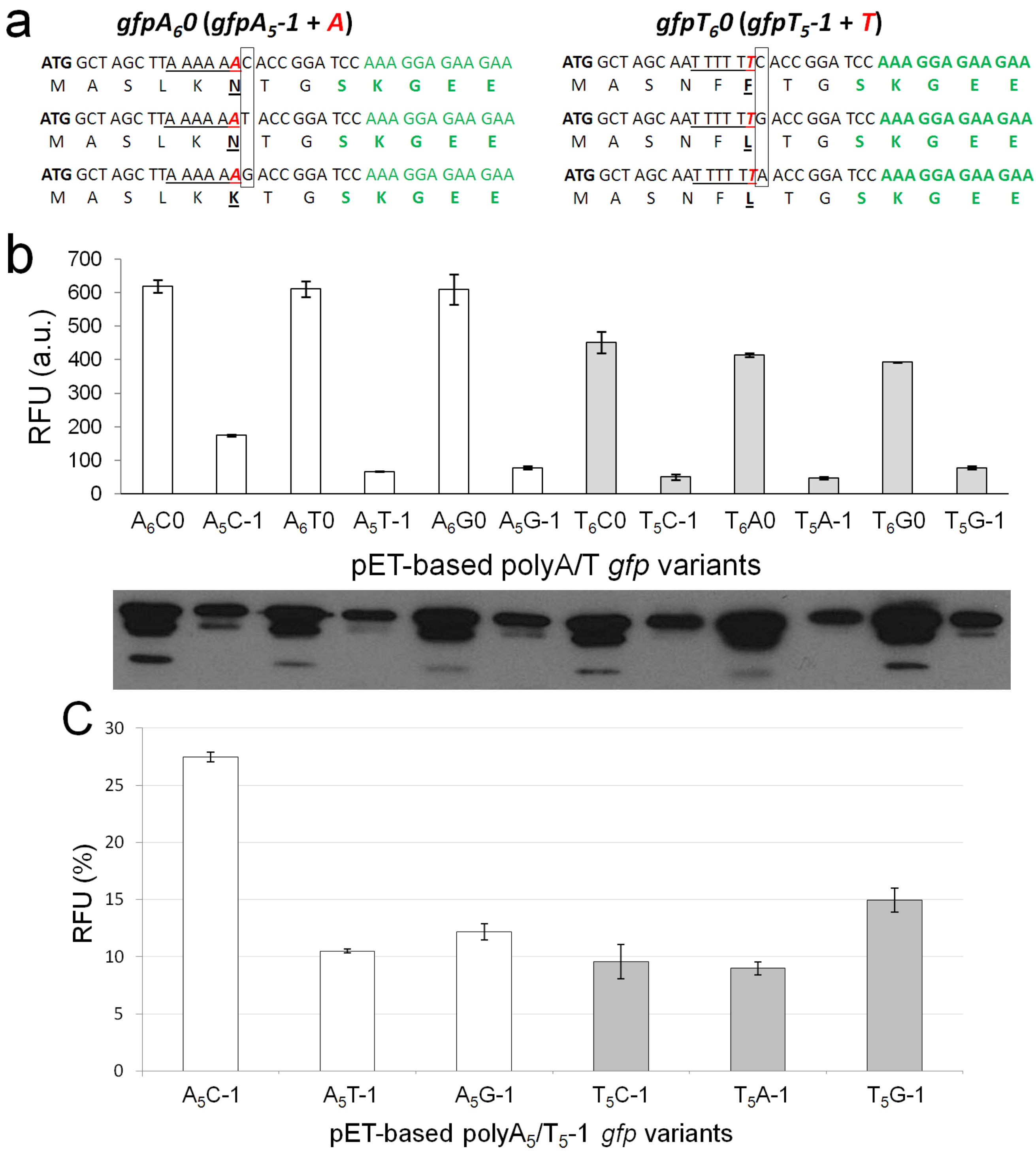

Figure S8. Effect of succeeding nucleotide on backward slippage efficiency of T7 RNAP. **a** Details of the DNA sequences of *gfp* fusion genes after single nucleotide insertion. DNA and amino acid compositions for *gfp*(A_6/T_6)*0* (frame 0) and *gfp*(A_5/T_5)*-1* (frame -1) after single nucleotide addition are identical. Inserted T/A nucleotides are marked in red and italicized. Nucleotides downstream of poly(A/T) are framed. In green, part of the wild-type *gfp* DNA/amino acid sequence is given. **b** Insertion slippage efficiency influenced by nucleotides following the poly(A_5/T_5) sequences reflected by GFP fluorescence (top panel) and expression (bottom panel). The reference frame 0 controls are shown to visualizes the lack of amino acid composition factor. Error bars represent standard deviation from at least three determinations. **c** Slippage efficiency after normalization to appropriate GFP frame 0 control.

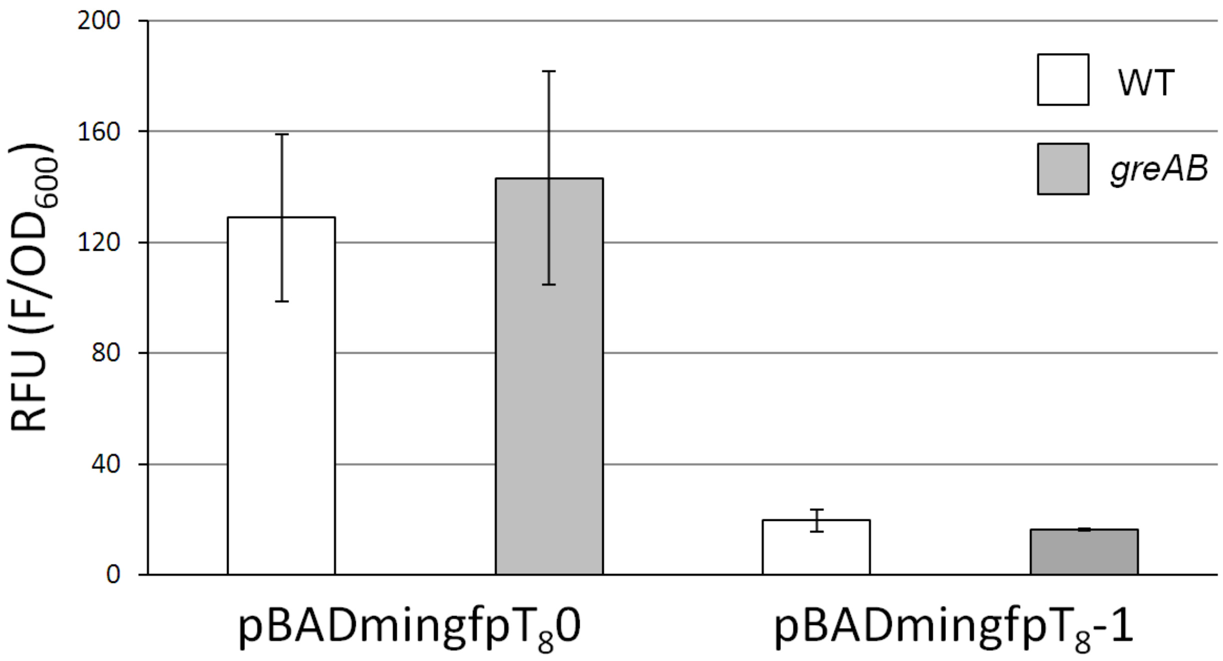

Figure S9. Bypass of the *gfp* deletion mutant gene by slippage in *E. coli* MC1061Δ*greAB* strain. MC1061 was used as an isogenic WT strain. Expression of *gfp* in- (*gfpT*$_8$*0*) and out-of-frame (*gfpT*$_8$*-1*) genes were induced with 0.1% L-arabinose for 1h, followed by fluorescence measurement. Error bars represent standard deviation from at least six determinations.

Table S1. Plasmids used in this study.

Plasmid name	Relevant feature(s)	Reference
**pACYC177**	P15 ori vector, Km^R, Amp^R	Chang and Cohen, 1973
**pACYC177araCN**	pACYC177 derivative with 3208 bp ApaLI DNA fragment of pBAD24N carrying *araC-P_{araBAD}-N* transcriptional unit inserted in FspI site, Km^R, Amp^S	This work
**pANTS**	Integrative vector with λ *attP* site, pBR322 ori, Amp^R	Posfai et al., 1994
**pANTSaraT7mboBΔA356**	pANTS derivative with *mboIIM2ΔA356* gene under control of P_{araBAD} and T7 phage promoters, excised from pBAD24/T7mboΔ and cloned between ClaI and HindIII sites	This work
**pBAD24**	arabinose inducible *araBAD* promoter, *araC*, pBR322 ori, Amp^R	Guzman et al., 1995
**pBAD24N**	pBAD24 derivative with PCR created λ phage N gene inserted into XbaI site,	This work
**pBAD24/T7mboΔ**	pBAD24 derivative carrying *mboIIM2ΔA356* gene excised with BglII-HindIII from pETmboIIMB.4 and inserted between BglII and HindIII sites, containing both, arabinose and T7 promoters,	This work
**pBAD24/T7mboWT**	pBAD24 derivative carrying *mboIIM2ΔA356* gene excised with BglII-HindIII from pETmboIIMB.3 and inserted between BglII and HindIII sites, containing both, arabinose and T7 promoters	This work
**pBAD24/T7mboWTlacI^q**	pBAD24/T7mboWT with the PCR-produced lacI^q gene cloned between XbaI-HindIII	This work
**pBADmboIIMB.4**	pBAD24 derivative carrying *mboIIM2ΔA356 gene* inserted between XbaI-HindIII, excised from pET24mboIIMB.4	This work
**pBADmingfpA$_8$-1**	Table S3	This work

**pBADmingfpA$_8$+1**	Table S3	
**pBADmingfpA$_7$-1**	Table S3	This work
**pBADmingfpA$_7$+1**	Table S3	This work
**pBADmingfpA$_6$0**	pBAD24 derivative with XbaI-HindIII subcloned fragment of pETgfpA$_6$0 carrying translational fusion of *gfp* gene with upstream A-rich fragment 5'- T$_2$A$_6$ (Table S3)	Wons et al., 2018
**pBADmingfpA$_6$-1**	Table S3	This work
**pBADmingfpA$_6$+1**	Table S3	This work
**pBADmingfpA$_5$0**	pBAD24 derivative with XbaI-HindIII subcloned fragment of pETgfpA$_5$0 carrying translational fusion *gfp* gene with upstream A-rich fragment 5'- T$_2$A$_5$ (Table S3)	Wons et al., 2018
**pBADmingfpA$_5$-1**	pBAD24 derivative with XbaI-HindIII subcloned fragment of pETminT2A5gfp-1 carrying of modified -1 frameshifted *gfp* gene, in translational fusion with upstream fragment of *mboIIM2ΔA356* gene (349-359 nt 5'-TTAAAAACACC) (Table S3)	This work
**pBADmingfpA$_5$+1**	Table S3	This work
**pBADmingfpA$_4$0**	pBAD24 derivative with XbaI-HindIII subcloned fragment of pETgfpA$_4$0 (Table S3)	Wons et al, 2018
**pBADmingfpA$_4$-1**	pBAD24 derivative with XbaI-HindIII subcloned fragment of pETmingfpA$_4$-1 (Table S3)	This work
**pBADmingfpA$_4$+1**	pBAD24 derivative with XbaI-HindIII subcloned fragment of pETmingfpA$_4$+1 (Table S3)	This work
**pBADmingfpA$_3$-1**	pBAD24 derivative with XbaI-HindIII subcloned fragment of pETmingfpA$_3$-1 (Table S3)	This work
**pBADmingfpA$_2$-1**	pBAD24 derivative with XbaI-HindIII subcloned fragment of pETmingfpA$_2$-1 (Table S3)	This work

**pBADmingfpA_2GA_2-1**	pBAD24 derivative with XbaI-HindIII subcloned fragment of pETmingfpA_2GA_2-1 (Table S3)	This work
**pBADmingfpA_1-1**	pBAD24 derivative with XbaI-HindIII subcloned fragment of pETmingfpA_1-1 (Table S3)	This work
**pBADmingfpT_8-1**	pBAD24 derivative with XbaI-HindIII subcloned fragment of pETmingfpT_8-1 (Table S3)	This work
**pBADmingfpT_8+1**	pBAD24 derivative with XbaI-HindIII subcloned fragment of pETmingfpT_8+1 (Table S3)	This work
**pBADmingfpT_7-1**	pBAD24 derivative with XbaI-HindIII subcloned fragment of pETmingfpT_7-1 (Table S3)	This work
**pBADmingfpT_7+1**	pBAD24 derivative with XbaI-HindIII subcloned fragment of pETmingfpT_7+1 (Table S3)	This work
**pBADmingfp$T_6$0**	Table S3	Wons et al., 2018
**pBADmingfpT_6-1**	pBAD24 derivative with XbaI-HindIII subcloned fragment of pETmingfpT_6-1 (Table S3)	This work
**pBADmingfpT_6+1**	pBAD24 derivative with XbaI-HindIII subcloned fragment of pETmingfpT_6+1 (Table S3)	This work
**pBADmingfp$T_5$0**	Table S3	Wons et al., 2018
**pBADmingfpT_5-1**	pBAD24 derivative with XbaI-HindIII subcloned fragment of pETmingfpT_5-1 (Table S3)	This work
**pBADmingfpT_5+1**	pBAD24 derivative with XbaI-HindIII subcloned fragment of pETmingfpT_5+1 (Table S3)	This work
**pBADmingfp$T_4$0**	Table S3	Wons et al., 2018
**pBADnutL**	pBAD24 derivative with minimal λ phage *nutL* antiterminator sequence (59 bp, Salstrom and Szybalski, 1979) inserted between EcoRI-XbaI sites	This work

**pBADnutMboWT**	pBAD24nutL derivative carrying *mboIIM2* gene inserted between XbaI-HindIII, excised from pET24mboIIMB.3	This work
**pBADnutMboΔA356**	pBAD24nutL derivative carrying *mboIIM2ΔA356* gene cloned between XbaI-HindIII, excised from pETmboIIMB.4	This work
**pBADnutMboΔA356Δ377**	pBADnutMboΔA356 derivative carrying 377 bp short variant of *mboIIM2ΔA356* gene cloned between XbaI-HindIII	This work
**pBADnutMboΔ378**	pBADnutMboWT derivative carrying 378 bp short variant of *mboIIM2* gene cloned between XbaI-HindIII	This work
**pBADmbo(A_5)mutL3 [ΔA356MutL3]**	pBADnutL derivative with XbaI-HindIII fragment carrying *mboIIM2* mutant from pET24mbo(A_5)mutL3 (Fig. 4)	This work
**pBADmbo(A_5)mutR3 [ΔA356MutR3]**	pBADnutL derivative with XbaI-HindIII fragment carrying *mboIIM2* mutant from pET24mbo(A_5)mutR3 (Fig. 4)	This work
**pBADmbo(A_5)mutLMR6 [ΔA356MutLMR6]**	pBADnutL derivative with XbaI-HindIII fragment carrying *mboIIM2* mutant from pET24mbo(A_5)mutLMR6 (Fig. 4)	This work
**pBADmboB+T372 [+T372]**	pBADnutL derivative with XbaI-HindIII fragment carrying *mboIIM2* mutant from pET24mboB+T372 (Fig. 4)	This work
**pBR322**	pMB1 ori, Amp^R Tc^R	Bolivar et al., 1977
**pET24a**	Used for translational fusion with RBS of gene *10* of T7, IPTG inducible T7 promoter, pBR ori, Km^R	Novagene
**pET24mboIIMB.3 [WT]**	pET24a derivative with the wild type *mboIIM2* methyltransferase gene cloned between NdeI-BamHI sites	Furmanek-Blaszk et al., 2009
**pET24mboIIMB.4 [ΔA356]**	pET24a derivative with frameshifting A356 deletion mutation in *mboIIM2*	Wons et al., 2015
**pET24remboIIMB.4**	pET24a derivative with *mboIIM2ΔA356* gene inserted between XbaI-HindIII sites, subcloned from pBADmboIIMB.4 plasmid	This work
**pET24mbo(A_5)mutL3**	pET24mboIIMB.4 derivative with double	Wons et al., 2015

	codon replacement, Leu 117 TTA codon to synonymous CTG and Lys 118 AAA to synonymous AAG	
**pET24mbo(A_5)mutR3**	pET24mboIIMB.4 derivative with triple codon replacement, Asn 121 AAC to AGC Ser, Phe 122 TTT to TCT Ser and Phe 123 TTT to TCT Ser	Wons et al., 2015
**pET24mbo(A_5)mutLMR6**	pET24mboIIMB.4 derivative with six replacements, Leu 117 TTA to synonymous CTG, Lys 118 AAA to synonymous AAG, Asn 121 AAC to AGC Ser codon, Phe 122 TTT to TCT Ser and Phe 123 TTT to TCT Ser	Wons et al., 2015
**pET24mboB+T372 (+T372)**	pET24mboIIMB.3 derivative with frameshifting T372 insertion mutation in *mboIIM2* (frame +1)	Wons et al., 2015
**pETmboBΔA356Δ562gfp-1**	pET24a derivative with 562 nt proximal part of *mboIIM2ΔA356Δ562* (-1 frameshift) and *gfp* genes (ΔA574, -1 frameshift)	Wons et al., 2015
**pETmingfpA_8-1**	Table S3	Wons et al., 2018
**pETmingfpA_8+1**	Table S3	Wons et al., 2018
**pETmingfpA_7-1**	pET24a derivative with BamHI/EcoRI PCR created *gfp* in frame -1 in translational fusion with upstream A-rich fragment 5'-T_2A_7 inserted between NheI-BamHI sites (Table S3)	This work
**pETmingfpA_7+1**		
**pETmingfp$A_6$0 (pETmingfpA_6C0)**	pET24a derivative with BamHI/EcoRI PCR created frame 0 *gfp* gene in translational fusion with upstream A-rich fragment 5'-T_2A_6 inserted between NheI-BamHI sites (Table S3)	Wons et al., 2018
**pETmingfpA_6G0**	pET24a derivative with BamHI/EcoRI PCR created frame 0 *gfp* gene in translational fusion with upstream A-rich fragment 5'-T_2A_6 inserted between NheI-BamHI sites (Fig. S7)	This work

**pETmingfpA_6T0**	pET24a derivative with BamHI/EcoRI PCR created frame 0 *gfp* gene in translational fusion with upstream A-rich fragment 5'-T_2A_6 inserted between NheI-BamHI sites (Fig. S7)	Wons et al., 2018
**pETmingfpA_6-1**	pET24a derivative with BamHI/EcoRI PCR created *gfp* in frame -1 in translational fusion with upstream A-rich fragment 5'-T_2A_6 inserted between NheI and BamHI sites (Table S3)	This work
**pETmingfp$A_5$0**	pET24a derivative with PCR created modified *gfp* gene in frame 0, in translational fusion with upstream A-rich fragment of *mboIIM2ΔA356* gene (349-360 nt 5'-TTAAAAAACACC) inserted between NheI and EcoRI sites (Table S3)	Wons et al., 2018
**pETmingfpA_5-1 (pETmingfpTTA_5-1) (pETmingfpA_5C-1)**	pET24a derivative with PCR created modified *gfp* gene -1 frameshifted, in translational fusion with upstream fragment of *mboIIM2ΔA356* gene (349-359 nt 5'-TTAAAAACACC) inserted between NheI and EcoRI sites (Table S3)	This work
**pETmingfpCTA_5-1**	Fig. S6a	This work
**pETmingfpTCA_5-1**	Fig. S6a	This work
**pETmingfpCCA_5-1**	Fig. S6a	This work
**pETmingfpGGA_5-1**	Fig. S6a	This work
**pETmingfpA_5+1 (pETmingfpTTA_5+1)**	Fig. S6a, Table S3	This work
**pETmingfpCCA_5+1**	Fig. S6a	This work
**pETmingfpA_5T-1**	Fig. S7a	This work
**pETmingfpA_5G-1**	Fig. S7a	This work
**pETmingfp$A_4$0**	Table S3	Wons et al., 2018
**pETmingfpA_4-1**	pETminT2A5gfp-1 derivative with alteration of single A on C (16 nt) 5'-TTAAAACCACC (Table S3)	This work

**pETmingfpA$_4$+1**	Table S3	This work
**pETmingfpA$_3$-1**	pETminT2A4gfp-1 derivative with alteration of single A on G (15 nt) 5'-TTAAAGCCACC (Table S3)	This work
**pETmingfpA$_2$-1**	pETminT2A3gfp-1 derivative with alteration of single A on G (14 nt) 5'-TTAACGCCACC (Table S3)	This work
**pETmingfpA$_1$-1**	pETmingfpA$_2$-1 derivative with alteration of single A on G (13 nt) 5'-TTAGCGCCACC (Table S3)	This work
**pETmingfpA$_2$GA$_2$-1**	pET24mingfpA$_5$-1 derivative with alteration of single A on G in the middle of As run (14 nt) 5'-TTAAGAACACC (Table S3)	This work
**pETmingfpT$_8$-1**	Table S3	This work
**pETmingfpT$_8$+1**	Table S3	This work
**pETmingfpT$_7$-1**	Table S3	This work
**pETmingfpT$_7$+1**	Table S3	This work
**pETmingfpT$_6$0**	Table S3	This work
**pETmingfpT$_6$-1**	Table S3	This work
**pETmingfpT$_6$+1**	Table S3	This work
**pETmingfpT$_5$-1 (pETmingfpAAT$_5$-1) (pETmingfpT$_5$C-1**	pET24a derivative with PCR created modified *gfp* gene -1frameshifted, in translational fusion with upstream fragment AATTTTTCACC) inserted between NheI and EcoRI sites (Table S3, Fig. S6a, Fig. S7a)	This work
**pETmingfpCAT$_5$-1**	Fig. S6a	This work
**pETmingfpACT$_5$-1**	Fig. S6a	This work
**pETmingfpCCT$_5$-1**	Fig. S6a	This work
**pETmingfpCGT$_5$-1**	Fig. S6a	This work
**pETmingfpGGT$_5$-1**	Fig. S6a	This work
**pETmingfpT$_5$A-1**	Fig. S7a	This work
**pETmingfpT$_5$G-1**	Fig. S7a	This work
**pETmingfpT$_5$+1 (pETmingfpAAT$_5$+1)**	Fig. 6a, Table S3	This work
**pETmingfpCCT$_5$+1**	Fig. S6a	This work

**pETmingfpT$_4$0**	Table S3	Wons et al., 2018
**pET24mboBΔA356+T371 [ΔA356+T371]**	pET24mboIIMB.4 derivative with -1 frameshifting A356 deletion and wild-type 0-frame restoring insertion T371 in *mboIIM2*	Wons et al., 2015
**pET24mboBwtΔ378**	pET24mboIIMB.3 derivative with proximal fragment of *mboIIM2* (378 nt) with BamHI sites on its distal terminus	Wons et al., 2015
**pET24mboBΔA356Δ377 [ΔA356Δ377]**	pET24mboIIMB.4 derivative with proximal fragment of *mboIIM2ΔA356* (377 nt) with BamHI sites on its distal terminus	Wons et al., 2015
**pGreenTIR**	IPTG inducible *lac* promoter with *gfp* from *Aequora victoria*, ori pUC, Amp^R	Miller and Lindow, 1995
**pUC18**	pUC ori, Amp^R	Yanisch-Perron et al., 1985

Table S2. List of oligonucleotides used in this study.

No	Name, forward, reverse	Sequence (5'-3')	Comment
1	2T4A (f)	TGGCTAGCTTAAAACCACCGGATCCA	Primers 1 and 42 were used to the reduction of the length of polyA region of pETmingfpA$_5$-1 plasmid series by alteration of single A on C (16 nt) 5'-TTAAAACCACC (NheI site underlined)
2	2T3A (f)	TGGCTAGCTTAAAGCCACCGGATCCA	Primers 2 and 42 were used to the reduction of the length of polyA region of pETmingfpA$_5$-1 plasmid series by alteration of single A on G (15 nt) 5'-TTAAAGCCACC (NheI site)
3	2T2A (f)	TGGCTAGCTTAACGCCACCGGATCCA	Primers 3 and 42 were used to the reduction of the length of polyA region of pETmingfpA$_5$-1 plasmid series by alteration of single A on C (14 nt) 5'-TTAACGCCACC (NheI site)
4	2T1A (f)	TGGCTAGCTTAGCGCCACCGGATCCA	Primers 4 and 41 were used to reduction of length polyA region in pETmingfpA$_5$-1 plasmid series by alteration of single A on G (13 nt) 5'-TTAGCGCCACC (NheI site)
5	4rrnT1 (f)	AATCTAGAAATTCTAGGCGTATCACGAGGCCCTTTCGTCTTC	Forward primer to PCR production of 4 × *rrnBT1-lacZ*$^+$ transcriptional unit from pRS415 plasmid (XbaI site)
6	6AL (r)	GTGTTTTTTAAGCTAGCCAT	Primers 6 and 7 were used to insert 6th A to A/T-rich region 5'-TTAAAAA by PCR mutagenization (underlined)
7	6AR (f)	CTTAAAAAACACCGGATCCAAG	
8	6TL (r)	GTGAAAAAATTGCTAGCCAT	Primers 8 and 9 were used to insert 6th T to A/T-rich region 5'-AATTTTT by PCR mutagenization
9	6TR (f)	GCAATTTTTTCACCGGATCCAAG	

			(underlined)
10	7AL (r)	GTGTTTTTTTAAGCTAGCCAT	Primers 10 and 11 were used to insert 7th A to A/T-rich region 5’-TTAAAAAA by PCR mutagenization (underlined)
11	7AR (f)	CTTAAAAAAACACCGGTCCAAG	
12	7TL (r)	GTGAAAAAAATTGCTAGCCAT	Primers 12 and 13 were used to insert 7th T to A/T-rich region 5’-AATTTTTT by PCR mutagenization (underlined)
13	7TR (f)	GCAATTTTTTTCACCGGTCCAAG	
14	ATT2gfp (f)	CCCCATGGTTGTTGAATTAGATGGTGATG	Primers 14 and 17 were used to create shorter version of *gfp* beginning from alternative AUU start codon (altered on AUG) without first 14 codons (NcoI site)
15	blaF (f)	GGATCATGTAACTCGCCTTGAT	Primers 15 and 16 complementary to Tn3 *bla* gene used as internal marker in RT-qPCR
16	blaR (r)	CCGGGAAGCTAGAGTAAGTAGT	
17	Gfpdown (r)	CAGTGCCAAGCTTGCATGCCT	Reverse primer with HidIII site to distal part of the *gfp* carrying on pGreenTIR plasmid (Miller and Lindow, 1997)
18	GfpTplusL (r)	GTG**A**AAAAGTTCTTCTCCTTTGGATCC	Primers 18 and 19 used to insert additional T into TTTT run of *gfp*-1 gene creating frame 0
19	GfpTplusR (f)	GAGAAGAACTTTTT**T**CACTGGAGTTGTCC	
20	InterF (f)	TAGCGACATTCAACAAACCATT	Primers 20 and 21 complementary to the middle part of *mboIIM2* gene used in RT-qPCR
21	InterR (r)	CTTGGATAAGGATAGAACCAAACA	
22	lacqF (f)	GGGAATTCGAATGGTGCAAAACCTTTCGC	Primers 22 and 23 used to lacI^{q} PCR production with EcoRI and HindIII sites (underlined)
23	lacqR (r)	CCAAGCTTGCGCTCACTGCCCGCTTTCC	
24	LacXba (r)	AATCTAGACGGGCAGACATGGCCTGCCCGGT	Riverse primer to *lacZ* reporter gene production by PCR (XbaI site)
25	Mbo2F (f)	AGGACTATGCCAAAGATGGTTAT	Primers 25 and 26 complementary to the proximal part of *mboII* gene used in RT-qPCR
26	Mbo2R (r)	CCAATTTGCCGTTCGGTTT	

27	minA5 (f)	ATGGCTAGCTTAAAAACACCGGATCCAAAGGAGAAGAAC	Primers 27 and 17 were used to create *gfp* fusion gene with 5’-TTAAAAA poly(AT)-rich sequence (NheI and BamHI sites)
28	minA5G (f)	ATGGCTAGCTTAA**G**AACACCGGATCCA	Forward primer to mutagenization of the polyA/T rich sequence by **G** insertion (NheI and BamHI sites)
29	minA5mbo (r)	GGGATCCGGTGTTTTTAAAATGGTTTGTTGAATG	Reverse primer to create distal deletion variant of *mboIIM2* gene (Δ359-822 bp) to *gfp* fusion partner (BamHI site)
30	minA5T (f)	ATGGCTAGCAATTTTTCACCGGATCCAAAGGAGAAG	Primers 30 and 17 were used to create *gfp* fusion gene with AATTTTT poly(AT)-rich sequence (NheI site)
31	Mmbo2 (f)	AGGACTATGCCAAAGATGGTTAT	Primers 31 and 32 complementary to the proximal part of *mboIM2* gene used in RT-qPCR
32	Mmbo2 (r)	CCAATTTGCCGTTCGGTTT	
33	Nend (r)	TCTAAGCTTCTAGATAAGAGGAATC	Primers 33 and 34 were used to created N antiterminator protein encoding gene of λ phage (XbaI site)
34	Nfor (f)	AAGTCTAGAAAGCTAACTAACTGACAGGAGA	
35	nutD (r)	CTAGAATGCTGCCCTTCTTCAGGGCTTAATTTTTAAGAGCGTCACCTTCAT	Pair of primers to constitute minimal 43 bp λ phage *nutL* antiterminator sequence (Hasan and Szybalski, 1986) after annealing to fit XbaI and EcoRI sites of pBAD24 vector
36	nutG (f)	AATTCATGAAGGTGACGCTCTTAAAAATTAAGCCCTGAAGAAGGGCAGCAT	
37	rrnF (f)	TCGGAATTACTGGGCGTAAAG	Primers 36 and 37 complementary to the set of *rrn* genes used as internal marker in RT-qPCR
38	rrnR (r)	CCTCCAGATCTCTACGCATTTC	
39	RT-mboB	GATGAATTGGTTCTATTCCAAAGATAAGTG	Primer for RT-PCR reaction of *mboIIM2ΔA356* mRNA pool (corresponding to residues 377-406)
40	StabF (f)	GTTTGGCAAGAATGTGAACGAG	Primers 40 and 41 complementary to the proximal part of *mboII* gene gene used in RT-qPCR
41	StabR (r)	TGTCTGGTATAATGGGTATTCATCTC	
42	T7ter (r)	GCTAGTTATTGCTCAGCGGTGG	Universal reverse primer to T7 terminator region of pET plasmid

			series
43	TGf (f)	GGAGAAGAACTGTTCACTGGAGTTG	Primers 43 and 44 were used to change T on G in TTTT run of *gfp* gene
44	TGr (r)	CAGTGAACAGTTCTTCTCCTTTGGATC	
45	TAAf (f)	GAAGAAATCCCAAGACGACTGA	Primers 45 and 46 complementary to the distal part of *mboIIM2* gene used in RT-qPCR
46	TAAr (r)	CGCTATATTGCTCCATTAACTCATAAC	
47	TAA2f (f)	GCAAGAATGGGTGGAATATACTAAA	Primers 47 and 48 complementary to the distal part of *mboIIM2* gene used in RT-qPCR
48	TAA2r (r)	CTTCTGGCATAATCGCTGAATG	
49	ZeroGFP (f)	TGGCTAGCTCGGCGCCACCGGATCCAAAGG	Primers 49 and 42 were used to create no-proximal A/T-gfp-1 fusion

Table S3. Sequences of constructs used in the experiment shown in Fig. 3.

Construct name	Gene	Data	Details of sequence modification
pBADgfpWT<br>pETgfpWT	*gfpWT*	original frame (0)	ATG AGT AAA GGA GAA GAA CTT TTC ACT GGA GTT GTC
		protein sequence	M S K G E E L F T G V V
pBADmingfp$A_4$0<br>pETmingfp$A_4$0	*gfp$A_4$0*	original frame (0)	ATG GCT AGC TTA AAA CCA CCG GAT CCA GGA GAA GAA
		protein sequence	M A S L K P P D P G E E
pBADmingfp$A_5$0<br>pETmingfp$A_5$0	*gfp$A_5$0*	original frame (0)	ATG GCT AGC TTA AAA ACA CCG GAT CCA AAA GGA GAA
		protein sequence	M A S L K T P D P K G E
pBADmingfp$A_6$0<br>pETmingfp$A_6$0	*gfp$A_6$0*	original frame (0)	ATG GCT AGC TTA AAA AAC ACC GGA TCC AAA GGA GAA
		protein sequence	M A S L K N T G S K G E
pBADmingfpA_1-1<br>pETmingfpA_1-1	*gfpA_1-1*	original frame (-1)	ATG GCT AGC TTA GCG CCA CCG GAT CCA AAG GAG AAG
		insertion type slippage (frame 0)	AAA GGA GAA
		protein sequence	M A S L A P P D P K G E
pBADmingfpA_2-1<br>pETmingfpA_2-1	*gfpA_2-1*	original frame (-1)	ATG GCT AGC TTA ACG CCA CCG GAT CCA AAG GAG AAG
		insertion type slippage (frame 0)	AAA GGA GAA
		protein sequence	M A S L T P P D P K G E
pBADmingfpA_2GA_2-1<br>pETmingfpA_2GA_2-1	*gfpA_2GA_2-1*	original frame (-1)	ATG GCT AGC TTA AGA ACA CCG GAT CCA AAG GAG AAG
		insertion type slippage (frame 0)	AAA GGA GAA
		protein sequence	M A S L R T P D P K G E
pBADmingfpA_3-1<br>pETmingfpA_3-1	*gfpA_3-1*	original frame (-1)	ATG GCT AGC TTA AAG CCA CCG GAT CCA AAG GAG AAG
		insertion type slippage (frame 0)	AAA GCC ACC GGA TCC AAA GGA GAA
		protein sequence	M A S L K A T G S K G E
pBADmingfpA_4-1<br>pETmingfpA_4-1	*gfpA_4-1*	original frame (-1)	ATG GCT AGC TTA AAA CCA CCG GAT CCA AAG GAG AAG
		insertion type slippage (frame 0)	ACC ACC GGA TCC AAA GGA GAA
		protein sequence	M A S L K T T G S K G E
pBADmingfpA_5-1<br>pETmingfpA_5-1	*gfpA_5-1*	original frame (-1)	ATG GCT AGC TTA AAA ACA CCG GAT CCA AAG GAG AAG
		insertion type slippage (frame 0)	AAC ACC GGA TCC AAA GGA GAA
		protein sequence	M A S L K N T G S K G E
pBADmingfpA_6-1<br>pETmingfpA_6-1	*gfpA_6-1*	original frame (-1)	ATG GCT AGC TTA AAA AAC ACC GGA TCC AAG GAG AAG
		insertion type slippage (frame 0)	AAA CAC CGG ATC CAA GGA GAA
		protein sequence	M A S L K K H R I Q G E
pBADmingfpA_7-1<br>pETmingfpA_7-1	*gfpA_7-1*	original frame (-1)	ATG GCT AGC TTA AAA AAA CAC CGG ATC CAG GAG AAG
		insertion type slippage (frame 0)	ACA CCG GAT CCA GGA GAA
		protein sequence	M A S L K K T P D P G E
pBADmingfpA_8-1<br>pETmingfpA_8-1	gfpA_8-1	original frame (-1)	ATG GCT AGC TTA AAA AAA ACC CGG ATC CAG GAG AAG
		insertion type slippage (frame 0)	AAC CCG GAT CCA GGA GAA
		protein sequence	M A S L K K N P D P G E
pBADmingfp$T_4$0<br>pETmingfp$T_4$0	*gfp$T_4$0*	original frame (0)	ATG GCT AGC AAT TTT CCA CCG GAT CCA GGA GAA GAA
		protein sequence	M A S N F P P D P G E E
pBADmingfp$T_6$0<br>pETmingfp$T_6$0	*gfp$T_6$0*	original frame (0)	ATG GCT AGC AAT TTT TTC ACC GGA TCC AAA GGA GAA
		protein sequence	M A S N F F T G S K G E
pBADmingfpT_4-1<br>pETminT_4gfp-1	*gfpT_4-1*	original frame (-1)	ATG GCT AGC AAT TTT GCA CCG GAT CCA AAG GAG AAG
		insertion type slippage (frame 0)	TGC ACC GGA TCC AAA GGA GAA
		protein sequence	M A S N F C T G S K G E
pBADmingfpT_5-1<br>pETmingfpT_5-1	*gfpT_5-1*	original frame (-1)	ATG GCT AGC AAT TTT TCA CCG GAT CCA AAG GAG AAG
		insertion type slippage (frame 0)	TTC ACC GGA TCC AAA GGA GAA
		protein sequence	M A S N F F T G S K G E
pBADmingfpT_6-1<br>pETmingfpT_6-1	*gfpT_6-1*	original frame (-1)	ATG GCT AGC AAT TTT TTC ACC GGA TCC AAG GAG AAG
		insertion type slippage (frame 0)	TTT CAC CGG ATC CAA GGA GAA
		protein sequence	M A S N F F H R I Q G E

pBADmingfpT$_7$-1<br>pETmingfpT$_7$-1	*gfpT$_7$-1*	original frame (-1)	ATG	GCT	AGC	AAT	TTT	TTT	CAC	CGG	ATC	CAG	GAG	AAG
		insertion type slippage (frame 0)							TCA	CCG	GAT	CCA	GGA	GAA
		protein sequence	M	A	S	N	F	F	S	P	D	P	G	E
pBADmingfpT$_8$-1<br>pETmingfpT$_8$-1	*gfpT$_8$-1*	original frame (-1)	ATG	GCT	AGC	AAT	TTT	TTT	TAC	CGG	ATC	CAG	GAG	AAG
		insertion type slippage (frame 0)							TTA	CCG	GAT	CCA	GGA	GAA
		protein sequence	M	A	S	N	F	F	L	P	D	P	G	E
pBADmingfpA$_4$+1<br>pETmingfpA$_4$+1	*gfpA$_4$+1*	original frame (+1)	ATG	GCT	AGC	TTA	AAA	CCA	CCG	GAT	CCA	AGG	AGA	AGA
		deletion type slippage (frame 0)					AAC	CAC	CGG	ATC	CAA	GGA	GAA	GAA
		protein sequence	M	A	S	L	N	H	R	I	Q	G	E	E
pBADmingfpA$_5$+1<br>pETmingfpA$_5$+1	*gfpA$_5$+1*	original frame (+1)	ATG	GCT	AGC	TTA	AAA	ACA	CCG	GAT	CCA	AGG	AGA	AGA
		deletion type slippage (frame 0)						CAC	CGG	ATC	CAA	GGA	GAA	GAA
		protein sequence	M	A	S	L	K	H	R	I	Q	G	E	E
pBADmingfpA$_6$+1<br>pETminAgfpA$_6$+1	*gfpA$_6$+1*	original frame (+1)	ATG	GCT	AGC	TTA	AAA	AAC	ACC	GGA	TCC	AGG	AGA	AGA
		deletion type slippage (frame 0)						ACA	CCG	GAT	CCA	GGA	GAA	GAA
		protein sequence	M	A	S	L	K	T	P	D	P	G	E	E
pBADmingfpA$_7$+1<br>pETmingfpA$_7$+1	*gfpA$_7$+1*	original frame (+1)	ATG	GCT	AGC	TTA	AAA	AAA	CAC	CGG	ATC	CAA	AGG	AGA
		deletion type slippage (frame 0)						AAC	ACC	GGA	TCC	AAA	GGA	GAA
		protein sequence	M	A	S	L	K	N	T	G	S	K	G	E
pBADmingfpA$_8$+1<br>pETmingfpA$_8$+1	*gfpA$_8$+1*	original frame (+1)	ATG	GCT	AGC	TTA	AAA	AAA	ACC	CGG	ATC	CAA	AGG	AGA
		deletion type slippage (frame 0)							CCC	GGA	TCC	AAA	GGA	GAA
		protein sequence	M	A	S	L	K	K	P	G	S	K	G	E
pBADmingfpT$_4$+1<br>pETmingfp T$_4$+1	*gfpT$_4$+1*	original frame (+1)	ATG	GCT	AGC	AAT	TTT	GCA	CCG	GAT	CCA	AGG	AGA	AGA
		deletion type slippage (frame 0)					TTG	CAC	CGG	ATC	CAA	GGA	GAA	GAA
		protein sequence	M	A	S	N	L	H	R	I	Q	G	E	E
pBADmingfpT$_5$+1<br>pETmingfpT$_5$+1	*gfpT$_5$+1*	original frame (+1)	ATG	GCT	AGC	AAT	TTT	TCA	CCG	GAT	CCA	AGG	AGA	AGA
		deletion type slippage (frame 0)						CAC	CGG	ATC	CAA	GGA	GAA	GAA
		protein sequence	M	A	S	N	F	H	R	I	Q	G	E	E
pBADmingfpT$_6$+1<br>pETmingfpT$_6$+1	*gfpT$_6$+1*	original frame (+1)	ATG	GCT	AGC	AAT	TTT	TTC	ACC	GGA	TCC	AGG	AGA	AGA
		deletion type slippage (frame 0)						TCA	CCG	GAT	CCA	GGA	GAA	GAA
		protein sequence	M	A	S	N	F	S	P	D	P	G	E	E
pBADmingfpT$_7$+1<br>pETmingfpT$_7$+1	*gfpT$_7$+1*	original frame (+1)	ATG	GCT	AGC	AAT	TTT	TTT	CAC	CGG	ATC	CAA	AGG	AGA
		deletion type slippage (frame 0)						TTC	ACC	GGA	TCC	AAA	GGA	GAA
		protein sequence	M	A	S	N	F	F	T	G	S	K	G	E
pBADmingfpT$_8$+1<br>pETmingfpT$_8$+1	*gfpT$_8$+1*	original frame (+1)	ATG	GCT	AGC	AAT	TTT	TTT	TAC	CGG	ATC	CAA	AGG	AGA
		deletion type slippage (frame 0)							ACC	GGA	TCC	AAA	GGA	GAA
		protein sequence	M	A	S	N	F	F	T	G	S	K	G	E

PolyA and polyT homopolymers are underlined and marked in blue. Right and left oriented red arrows indicate direction and the most likely site of slippage (inserted or deleted A/T nucleotide is marked in red). The original *gfp* nucleotide and amino acid sequence is given in green.

Table S4. Error frequency and sequencing depth of *mboIIM2Δ356* mRNA pool (1-376 nt) generated by *E. coli* RNAP.

base number	misincorporation rate	Insertions rate (%)	deletions rate (%)	homopolymer length	sequencing depth
0	0.0	0.0	0.0	1	5432
1	0.0	0.0	0.0	1	5493
2	0.10777797736662474	0.0	0.0	1	5567
3	0.17911517105498836	0.0	0.0	2	5583
4	0.12520121624038635	0.0	0.0	2	5591
5	0.1781895937277263	0.0	0.0	1	5612
6	0.26666666666666666	0.0	0.0	1	5625
7	0.17743080198722497	0.0	0.0	2	5636
8	0.15943312666076173	0.0	0.0	2	5645
9	0.1412928293889085	0.0	0.0	1	5662
10	0.08823010411152285	0.0	0.0	1	5667
11	0.2573781743308167	0.8236101578586137	0.051475634866163356	1	5828
12	0.06855184233076264	0.0	0.08568980291345331	6	5835
13	0.0678886625933469	0.0	0.0	6	5892
14	0.0676818950930626	0.0	0.0	6	5910
15	0.5900202292650034	0.0	0.0	6	5932
16	0.08424599831508003	0.0	0.0	6	5935
17	0.050462573591253154	0.0	0.0	6	5945
18	0.03354579000335458	0.0	0.01677289500167729	3	5962
19	0.10045203415369162	0.016742005692281934	0.0	3	5973
20	0.15030060120240482	0.0	0.0	3	5988
21	0.21670278379729954	0.0	0.0	1	5999
22	0.2164141834526386	0.0	0.0	1	6007
23	0.19890601690701143	0.0	0.0	1	6033
24	0.06609385327164573	0.0	0.0	1	6052
25	0.27905449770190416	0.0	0.0	1	6092
26	0.17889087656529518	0.0	0.0	2	6149
27	0.2432300956705043	0.0	0.0	2	6167
28	0.11273957158962795	0.0	0.0	1	6209
29	0.24142926122646063	0.0	0.0	1	6213
30	0.016066838046272493	0.0	0.0	2	6224
31	0.144555091551558	0.0	0.0	2	6226
32	0.12836970474967907	0.0	0.0	1	6232
33	0.0641025641025641	0.0	0.0	1	6240
34	0.04803073967339097	0.0	0.0	1	6246
35	0.11061946902654868	0.0	0.0	1	6328
36	0.12554927809165098	0.0	0.0	1	6372
37	0.09389671361502347	0.0	0.0	2	6390
38	0.09383797309978105	0.0	0.0	2	6394
39	0.10911925175370225	0.0	0.0	1	6415
40	0.10905125408942204	0.0	0.0	2	6419
41	0.24879489970455604	0.0	0.0	2	6431
42	0.12410797393732546	0.0	0.0	1	6446
43	0.4186695611722748	0.0	0.0	1	6449
44	0.278508432616432	0.0	0.0	1	6463
45	0.15363343063450607	0.0	0.0	3	6509
46	0.5661820964039787	0.0	0.0	3	6535
47	0.30353619669145543	0.0	0.0	3	6589
48	0.10607667828458857	0.0	0.0	2	6599
49	0.2118003025718608	0.0	0.0	2	6610
50	0.10570824524312897	0.0	0.0	1	6622

51	0.15067048365225252	0.0	0.0	2	6637
52	0.045112781954887216	0.0	0.0	2	6650
53	0.13531799729364005	0.0	0.0	1	6651
54	0.1799370220422852	0.0	0.0	1	6669
55	0.2543006731488407	0.0	0.0	1	6685
56	0.371415837171297	0.029713266973703762	0.0	1	6731
57	0.11823825007389892	0.0	0.0	1	6766
58	0.22225514891094977	0.0	0.0	1	6749
59	0.25129342202512933	0.0	0.0	1	6765
60	0.1622897609914429	0.0	0.0	1	6778
61	0.1033210332103321	0.0	0.0	1	6775
62	0.07370283018867925	0.0	0.0	1	6784
63	0.16032648301996794	0.0	0.0	1	6861
64	0.08748906386701663	0.0	0.029163021289005542	2	6858
65	0.07290755322251385	0.043744531933508315	0.0	2	6858
66	0.13108068744538304	0.0	0.0	1	6866
67	0.14547570555717193	0.0	0.014547570555717195	2	6874
68	0.1606308411214953	0.0	0.0	2	6848
69	0.145602795573675	0.0	0.0	1	6868
70	0.10195164579085349	0.0	0.014564520827264782	2	6866
71	0.07279079924297568	0.0	0.0	2	6869
72	0.07271669575334497	0.0	0.014543339150668994	1	6876
73	0.11614401858304298	0.0	0.0	1	6888
74	0.043834015195791935	0.0	0.0	2	6844
75	0.1169248757673195	0.0	0.0	2	6842
76	0.23238925199709515	0.0	0.0	1	6885
77	0.20339968037193082	0.0	0.0	1	6883
78	0.10208546011375237	0.0	0.0	2	6857
79	0.21929824561403508	0.0	0.0	2	6840
80	0.4014869888475836	0.0	0.0	1	6725
81	0.10460251046025104	0.0	0.0	2	6692
82	0.08982035928143713	0.0	0.0	2	6680
83	0.07485029940119761	0.0	0.0	2	6680
84	0.14956625785222855	0.014956625785222853	0.0	2	6686
85	0.14985763524651582	0.02997152704930316	0.0	1	6673
86	0.10466507177033493	0.0	0.0	4	6688
87	0.44822949350067237	0.0	0.0	4	6693
88	0.16462137084705178	0.0	0.0	4	6682
89	0.12012012012012012	0.015015015015015015	0.0	4	6660
90	0.10578812150521386	0.0	0.0	2	6617
91	0.13574660633484162	0.0	0.0	2	6630
92	0.372150721042022	0.0	0.0	1	6449
93	0.01551590380139643	0.0	0.0	1	6445
94	0.12412723041117144	0.0	0.01551590380139643	2	6445
95	0.09283614420547734	0.0	0.0	2	6463
96	0.06171887054466903	0.0	0.0	2	6481
97	0.04620360388110272	0.0	0.0	2	6493
98	0.1835142988224499	0.0	0.0	2	6539
99	0.16915269875442104	0.0	0.0	2	6503
100	0.061919504643962855	0.0	0.0	1	6460
101	0.09233610341643582	0.0	0.0	1	6498
102	0.10802469135802469	0.030864197530864196	0.0	1	6480
103	0.20046260601387816	0.0	0.0	1	6485
104	0.07704160246533129	0.0	0.0	1	6490
105	0.16671718702637162	0.0	0.0	1	6598
106	0.2419476788144564	0.0	0.0	2	6613

107	0.22461814914645103	0.0	0.0	2	6678
108	0.059907143926913285	0.0	0.014976785981728321	3	6677
109	0.2546434991012583	0.0	0.0	3	6676
110	0.2843885645861398	0.0	0.0	3	6681
111	0.13471037269869782	0.0	0.0	1	6681
112	0.13617793917385385	0.0	0.0	1	6609
113	0.07555152614082805	0.0	0.0	1	6618
114	0.10575615651911163	0.0	0.030216044719746184	2	6619
115	0.25594700391448355	0.0	0.0	2	6642
116	0.39334341906202724	0.0	0.0	2	6610
117	0.07554011179936546	0.0	0.0	2	6619
118	0.10554885404101327	0.0	0.0	1	6632
119	0.07548309178743962	0.0	0.0	1	6624
120	0.13493253373313344	0.0	0.0	1	6670
121	0.14940983116689077	0.0	0.0	1	6693
122	0.17841213202497772	0.0	0.01486767766874814	1	6726
123	0.13432835820895522	0.0	0.0	1	6700
124	0.10439970171513796	0.0	0.0	1	6705
125	0.13434841021047916	0.0	0.0	3	6699
126	0.08931229532598987	0.0	0.0	3	6718
127	0.20830233596191042	0.014878738282993602	0.0	3	6721
128	0.10405827263267431	0.0	0.0	1	6727
129	0.04448398576512455	0.0	0.0	1	6744
130	0.3250591016548463	0.0	0.0	1	6768
131	0.29616466755516063	0.0	0.0	2	6753
132	0.3698224852071006	0.0	0.0	2	6760
133	0.04439183190292986	0.0	0.0	1	6758
134	0.07400828892835998	0.0	0.0	1	6756
135	0.01483239394838327	0.0	0.0	1	6742
136	0.3557663800770827	0.0	0.0	1	6746
137	0.17673048600883653	0.0	0.0	1	6790
138	0.13250883392226148	0.0	0.0	2	6792
139	0.15135462388375964	0.0	0.0	2	6607
140	0.06047777441790142	0.0	0.0	1	6614
141	0.09140767824497258	0.0	0.0	3	6564
142	0.18312223409125591	0.0	0.0	3	6553
143	0.25820170109356017	0.0	0.0	3	6584
144	0.25757575757575757	0.0	0.0	1	6600
145	0.19938650306748468	0.0	0.0	1	6520
146	0.1645228836374514	0.0	0.0	1	6686
147	0.074360499702558	0.0	0.0	1	6724
148	0.13131018383425735	0.0	0.0	1	6854
149	0.2455582839809331	0.0	0.01444460494005489	1	6923
150	0.23377337733773376	0.0	0.01375137513751375	1	7272
151	0.013044612575006522	0.0	0.013044612575006522	1	7666
152	0.16432815067627354	0.0	0.012640626975097966	1	7911
153	0.19540791402051783	0.0	0.012212994626282364	1	8188
154	0.10813408626697105	0.0	0.0	3	8323
155	0.14650481372959395	0.0	0.0	3	9556
156	0.18757815756565235	0.0	0.0	3	9596
157	0.30183180682764366	0.010407993338884263	0.010407993338884263	2	9608
158	0.23824321524756578	0.010358400662937643	0.0	2	9654
159	0.2767811378780113	0.0	0.02050230650948232	2	9755
160	0.32766741757116524	0.0	0.0	2	9766
161	0.417940876656473	0.010193679918450561	0.010193679918450561	1	9810
162	0.22267206477732793	0.0	0.010121457489878543	1	9880

163	0.14370765756518167	0.0	0.0	1	9742
164	0.1947519475194752	0.0	0.01025010250102501	1	9756
165	0.1328156926849203	0.0	0.02043318348998774	1	9788
166	0.23459812321501428	0.0	0.0	2	9804
167	0.15292078703231726	0.0	0.0	2	9809
168	0.21426385062748698	0.0	0.0	1	9801
169	0.12213740458015268	0.010178117048346055	0.0	1	9825
170	0.2537555826228177	0.0	0.0	1	9852
171	0.06089515883487262	0.0	0.0	1	9853
172	0.22235698403072568	0.0	0.0	1	9894
173	0.4336425978217023	0.01008471157724889	0.0	1	9916
174	0.1708027730332563	0.0	0.0	1	9953
175	0.14054813773717498	0.0	0.0	1	9961
176	0.21048411346096021	0.0	0.0	1	9977
177	0.10943095901313173	0.0	0.019896538002387585	1	10052
178	0.11918951132300357	0.0	0.0	2	10068
179	0.16052974816895757	0.0	0.0	2	9967
180	0.1404494382022472	0.0	0.0	1	9968
181	0.12153129430828438	0.0	0.0202552157180474	1	9874
182	0.19234662887224133	0.0	0.020247013565499086	2	9878
183	0.2225144128653788	0.0	0.0	2	9887
184	0.060410793395086586	0.0	0.02013693113169553	1	9932
185	0.20110608345902461	0.0	0.0	1	9945
186	0.2004208838560978	0.0	0.0	1	9979
187	0.14042126379137412	0.0	0.0	1	9970
188	0.25937749401436555	0.0	0.0	1	10024
189	0.5355548943766736	0.0	0.0	1	10083
190	0.3245203779707932	0.0	0.009544716999140975	1	10477
191	0.24799694772987407	0.0	0.0	1	10484
192	0.21706304265760665	0.0	0.0	1	10596
193	0.10370510040539267	0.0	0.028283209201470726	3	10607
194	0.1791269916093146	0.0	0.018855472800980485	3	10607
195	0.14149608527497406	0.0	0.0	3	10601
196	0.150929157626639	0.0	0.018866144703329875	1	10601
197	0.07475238273219958	0.0	0.018688095683049896	1	10702
198	0.30711959050721266	0.0	0.009306654257794323	2	10745
199	0.13897896784953212	0.0	0.0	2	10793
200	0.11033468186833395	0.0	0.0	4	10876
201	0.08269018743109151	0.0	0.0	4	10884
202	0.13777900248002206	0.0	0.0	4	10887
203	0.11891694109037688	0.0	0.0	4	10932
204	0.336180265309831	0.018171906232963837	0.0	1	11006
205	0.5376344086021506	0.0	0.0	3	10974
206	0.3095411507647487	0.0	0.0	3	10984
207	0.1543770432255721	0.0	0.0	3	11012
208	0.2890956725991508	0.0	0.009034239768723463	2	11069
209	0.4527755139002083	0.0	0.0	2	11043
210	0.37181303116147313	0.0	0.0	1	11296
211	0.11337868480725624	0.0	0.0	2	11466
212	0.078003120124805	0.0	0.0	2	11538
213	0.2338877338877339	0.0	0.0	1	11544
214	0.11265164644714037	0.0	0.0	2	11540
215	0.06828269033799932	0.0	0.0	2	11716
216	0.19609514877653678	0.0	0.0	1	11729
217	0.18468770987239758	0.0	0.0167897918065816	1	11912
218	0.17598256934551246	0.008380122349786308	0.0	1	11933

219	0.3311806590495115	0.0	0.0	1	12078
220	0.09878169245966416	0.0	0.0	2	12148
221	0.07375235597803818	0.0	0.0	2	12203
222	0.3477838887091556	0.0	0.0	1	12364
223	0.31510058980366806	0.0	0.016159004605316312	1	12377
224	0.08901116685547823	0.0	0.0	1	12358
225	0.16839066634592254	0.024055809477988934	0.0	1	12471
226	0.23287561230225648	0.0	0.02409058058299205	4	12453
227	0.21665864227250844	0.0	0.0	4	12462
228	0.12041422493377217	0.0	0.0	4	12457
229	0.10441767068273093	0.0	0.0	4	12450
230	0.08032128514056225	0.0	0.0	4	12450
231	0.07211538461538462	0.0	0.0	4	12480
232	0.09578544061302681	0.0	0.0	4	12528
233	0.24736674114267473	0.0	0.0	4	12532
234	0.11937922801432552	0.0	0.0	2	12565
235	0.22123893805309736	0.0	0.0	2	12656
236	0.2526847757422615	0.015792798483891344	0.0	1	12664
237	0.04013163175214704	0.0	0.0	2	12459
238	0.08043111075363951	0.0	0.0	2	12433
239	0.3281312525010004	0.008003201280512205	0.0	1	12495
240	0.33235736329825116	0.0	0.0	2	12637
241	0.32436708860759494	0.0	0.0	2	12640
242	0.2765705254839984	0.0	0.0	1	12655
243	0.07118000632711168	0.0	0.007908889591901298	3	12644
244	0.023805745119822252	0.0	0.0	3	12602
245	0.07922674694977025	0.0	0.0	3	12622
246	0.1741195092995647	0.0	0.0	2	12635
247	0.14250653154936269	0.0	0.0	2	12631
248	0.20485345099275132	0.0	0.02363693665300977	2	12692
249	0.29346446700507617	0.0	0.0	2	12608
250	0.26362038664323373	0.0	0.0	2	12518
251	0.09613843935266785	0.0	0.0	2	12482
252	0.24819855884707767	0.0	0.016012810248198558	1	12490
253	0.14414991591254905	0.0	0.00800832866180828	1	12487
254	0.14306151645207438	0.0	0.0	1	12582
255	0.07940919558484873	0.0	0.0	2	12593
256	0.08720469319803392	0.0	0.0	2	12614
257	0.10292138389676193	0.0	0.0	1	12631
258	0.37162963548667666	0.015814027041986242	0.015814027041986242	1	12647
259	0.1643578304766377	0.0	0.0	1	12777
260	0.20344287949921755	0.0	0.0	1	12780
261	0.303975058456742	0.0	0.0	2	12830
262	0.20918881227241032	0.038738668939335245	0.0	2	12907
263	0.17066170196260957	0.0	0.023272050267628578	3	12891
264	0.14769900497512436	0.0	0.0	3	12864
265	0.17890479153702551	0.0	0.0	3	12856
266	0.03892262182780632	0.0	0.0	2	12846
267	0.062174555063340325	0.0	0.0	2	12867
268	0.13944840409048653	0.0	0.0	1	12908
269	0.2580057671877371	0.0	0.0	1	13178
270	0.2501705708437571	0.0	0.015161852778409523	2	13191
271	0.3628666465074085	0.0	0.0	2	13228
272	0.12106537530266344	0.0	0.007566585956416465	2	13216
273	0.10607667828458857	0.0	0.0	2	13198
274	0.06825420900955559	0.0	0.0	1	13186

275	0.16638935108153077	0.007563152321887763	0.0	1	13222
276	0.09844755774327907	0.0	0.0	2	13205
277	0.06811473548777719	0.3935518050404904	0.0	2	13213
278	0.06796043192630069	0.0	0.43796722796949333	7	13243
279	0.045184125310640866	0.0	0.015061375103546954	7	13279
280	0.03758550702848981	0.0	0.0	7	13303
281	0.03745598921267511	0.0	0.0	7	13349
282	0.059772863120143446	0.0	0.0	7	13384
283	0.04484640107631362	0.02242320053815681	0.0	7	13379
284	0.06747132468700802	0.0	0.0	7	13339
285	0.20964360587002098	0.0	0.0	1	13356
286	0.06769971415676246	0.0	0.0	1	13294
287	0.20261143628995948	0.0075041272699984994	0.0	1	13326
288	0.03002101471029721	0.0	0.0	1	13324
289	0.1947565543071161	0.0	0.0	1	13350
290	0.2991325157044571	0.0	0.0	1	13372
291	0.06732495511669659	0.0	0.007480550568521843	2	13368
292	0.08975989228812925	0.007479991024010771	0.0	2	13369
293	0.1945379723157501	0.0	0.0	1	13365
294	0.8727435476652246	0.0	0.0	1	13406
295	0.23094688221709006	0.0	0.0	3	13423
296	0.17925162446784673	0.0	0.0	3	13389
297	0.23193176717043246	0.0	0.0	3	13366
298	0.06004202942059442	0.0	0.0	1	13324
299	0.17285435142041183	0.0	0.007515406583496168	2	13306
300	0.1653762309253552	0.0	0.0	2	13303
301	0.11281588447653429	0.0	0.0	1	13296
302	0.08273787138021813	0.0	0.0	1	13295
303	0.496054114994363	0.0	0.0	1	13305
304	0.11263798152737103	0.0	0.0075091987684914015	2	13317
305	0.16495463747469447	0.0	0.0	2	13337
306	0.08267568583239383	0.0	0.01503194287861706	1	13305
307	0.46585017657224437	0.0	0.015027425050717559	1	13309
308	0.12803133001958125	0.0	0.0	1	13278
309	0.1958421211208195	0.0	0.0	1	13276
310	0.03773015393902807	0.0	0.0	1	13252
311	0.11319900384876612	0.007546600256584409	0.0	1	13251
312	0.0830188679245283	0.007547169811320755	0.0	1	13250
313	0.20386590154032014	0.0	0.007550588945937783	5	13244
314	0.1815568499886527	0.0	0.007564868749527195	5	13219
315	0.24222239043221555	0.0	0.0	5	13211
316	0.20428236362260724	0.0	0.0	5	13217
317	0.0908609070947225	0.0	0.0	5	13207
318	0.18176310209027569	0.0	0.015146925174189639	1	13204
319	0.11389521640091116	0.0	0.022779043280182234	1	13170
320	0.07594167679222358	0.0	0.030376670716889428	3	13168
321	0.07598206823189728	0.0	0.015196413646379454	3	13161
322	0.03802281368821293	0.0	0.015209125475285171	3	13150
323	0.09889691898060098	0.0	0.038037276531000384	3	13145
324	0.06846709775580068	0.0	0.015214910612400151	3	13145
325	0.04567252797442339	0.0	0.0	3	13137
326	0.13714285714285715	0.0	0.0	1	13125
327	0.06874952257275992	0.0	0.0	1	13091
328	0.2137894174238375	0.0	0.0	1	13097
329	0.17551892551892553	0.0	0.0	1	13104
330	0.2137894174238375	0.0	0.0	1	13097

331	0.22208607750038292	0.0	0.0	1	13058
332	0.10732904017172647	0.0	0.0	1	13044
333	0.06146281499692685	0.0	0.0	1	13016
334	0.21511985248924403	0.0	0.0	2	13016
335	0.13065867343017445	0.0	0.0	2	13011
336	0.20754862018602505	0.007686985932815743	0.0	1	13009
337	0.20006155740227763	0.0	0.007694675284702985	2	12996
338	0.20778820994305064	0.0	0.0	2	12994
339	0.06928406466512702	0.0	0.0	1	12990
340	0.04625346901017577	0.0	0.0	3	12972
341	0.13122346584330374	0.0	0.0	3	12955
342	0.8490930142802007	0.0	0.0	3	12955
343	0.1621371216800494	0.0	0.0077208153180975915	2	12952
344	0.1857441374506617	0.0	0.0	2	12921
345	0.0620106968452058	0.0	0.0	1	12901
346	0.3100294527980158	0.0	0.023252208959851185	4	12902
347	0.2793729629054788	0.0	0.0	4	12886
348	0.22524271844660193	0.0	0.0	4	12875
349	0.1245136186770428	0.0622568093385214	0.0	4	12850
350	0.06234899851921129	0.0	0.02338087444470423	5	12831
351	0.1248829222603809	0.0	0.0	5	12812
352	0.12486343062275637	0.0	0.0	5	12814
353	0.14841431026402124	0.0	0.0	5	12802
354	0.39865551473462046	0.0	0.0	5	12793
355	0.13353232267693033	0.0	0.0	1	12731
356	0.6853631636993855	0.0	0.0	1	12694
357	0.1340059908560618	0.0	0.0	2	12686
358	0.18943878759175942	0.0	0.0	2	12669
359	0.07115186971302079	0.0	0.023717289904340265	3	12649
360	0.19780045889706463	0.0	0.0	3	12639
361	0.7918910357934749	0.0	0.0	3	12628
362	0.1346641318124208	0.7525348542458808	0.015842839036755384	1	12624
363	0.5231037489102005	0.0	0.15059047317111834	7	12617
364	0.356718192627824	0.0	0.0	7	12615
365	0.27762354247640203	0.0	0.0	7	12607
366	0.12707489476610276	0.0	0.0	7	12591
367	0.08753780041381505	0.0	0.0	7	12566
368	0.15947691571645004	0.0	0.0	7	12541
369	0.13669990350595046	0.0	0.0	7	12436
370	0.07270377251797398	0.0	0.008078196946441553	2	12379
371	0.14572538860103626	0.0	0.008095854922279794	2	12352
372	0.14574898785425103	0.0	0.0	1	12350
373	0.18656716417910446	0.0	0.0	2	12328
374	0.2760412438093692	0.0	0.0	2	12317
375	0.3810604832171234	0.0	0.04053834927841738	1	12334
376	0.29149797570850206	0.0	0.016194331983805668	1	12350
377	0.6251014775125833	0.008118201006656925	0.040591005033284625	1	12318
378	0.12182246406237311	0.0	0.008121497604158206	1	12313
379	0.29251645405054033	0.0	0.032501828227837815	2	12307
380	0.12205044751830757	0.0	0.0	2	12290
381	0.15481137456204677	0.008147967082212989	0.0	1	12273
382	0.3182374541003672	0.0	0.02447980416156671	1	12255
383	0.15516537362188648	0.0	0.016333197223356473	1	12245
384	0.29445444135449045	0.0	0.3108130214297399	3	12226
385	0.31167979002624674	0.0	0.03280839895013123	3	12192
386	0.1972062448644207	0.0	0.0	3	12170

387	0.20621958261156478	0.0	0.016497566608925183	2	12123
388	0.3854210305823209	0.0	0.0	2	11935
389	0.16986580601324955	0.0	0.02547987090198743	2	11774
390	0.16258771179188775	0.017114495978093443	0.017114495978093443	2	11686
391	0.1371977362373521	0.0	0.034299434059338024	1	11662
392	0.07776049766718507	0.0	0.017280110592707794	1	11574
393	0.22471910112359553	0.017286084701815037	0.0	1	11570
394	0.06148981026001405	0.0	0.026352775825720307	2	11384
395	0.3188097768331562	0.017711654268508677	0.0	2	11292
396	0.3003823047515019	0.0	0.01820498816675769	2	10986
397	0.2552773686794305	0.0	0.0	2	10185
398	0.12208769966425882	0.0	0.0	2	9829
399	0.12050832602979841	0.010955302366345312	0.0	2	9128
400	0.17724603965880137	0.0	0.011077877478675086	2	9027
401	0.20273694880892043	0.025342118601115054	0.0	2	7892
402	0.11687363038714389	0.0	0.029218407596785973	1	6845

Additional references

Bolivar, F., Rodriguez, R.L., Greene, P.J., Betlach, M.C., Heyneker, H.L., Boyer, H.W., Crosa, J.H., Falkow, S. (1977) Construction and characterization of new cloning vehicles. II. A multipurpose cloning system. Gene **2**:95-113.

Chang A.C.Y., and Cohen, S.N. (1978). Construction and characterization of amplifiable multicopy DNA cloning vehicles derived from the P15A cryptic miniplasmid. J. Bacteriol. 134: 1141–1156.

Guzman, L.M., Belin, D., Carlson, M.J., Beckwith, J. (1995) Tight regulation, modulation, and high-level expression by vectors containing the arabinose P_{BAD} promoter. J. Bacteriol. **177**:4121-4130.

Hasan, N, Szybalski, W. (1986) Boundaries of the nutL antiterminator of coliphage lambda and effects of mutations in the spacer region between *box*A and *box*B. Gene **50**: 87-96.

Miller, W.G., S.E. Lindow. (1997) An improved GFP cloning cassette designed for prokaryotic transcriptional fusions. Gene **191**:149-153.

Posfai,G., Koob,M., Hradecna,Z., Hasan,N., Filutowicz,M. and Szybalski,W. (1994) *In vivo* excision and amplification of large segments of the *Escherichia coli* genome. Nucleic Acids Res., **22**: 2392-2398.

Wons, E., Furmanek-Blaszk, B., Sektas, M. (2015) RNA editing by T7 RNA polymerase bypasses InDel mutations with high efficiency causing unexpected phenotypic changes. Nucleic Acids Res. **43**: 3950–3963.

Wons, E., Koscielniak, D., Szadkowska, M., Sektas M. (2018) Evaluation of GFP reporter utility for analysis of transcriptional slippage during gene expression. Microb. Cell Fact. **17**:150.

Yanisch-Perron C., Vieira C., Messing J. (1985) Improved M13 phage cloning vectors and host strains: nucleotide sequences of the M13mp18 and pUC19 vectors. Gene **33**:103-119.
